# Epidermal METTL1‐Mediated m7G Modification Drives Psoriatic Inflammation by Stabilizing *Bdkrb1* and Orchestrating Neutrophil Recruitment

**DOI:** 10.1002/advs.75970

**Published:** 2026-06-09

**Authors:** Chang Zhang, Jiayi Lu, Sihao Yan, Yirui Wang, Zhuo Li, Weiwei Chen, Yang Han, Ziyan Zhang, Yafen Yu, Qi Zhen, Liangdan Sun

**Affiliations:** ^1^ Department of Dermatology the First Affiliated Hospital of Anhui Medical University Hefei China; ^2^ Institute of Dermatology Anhui Medical University Hefei Anhui China; ^3^ Key Laboratory of Dermatology (Anhui Medical University) Ministry of Education Hefei Anhui China; ^4^ Collaborative Innovation Center of Complex and Severe Skin Disease Anhui Medical University Hefei Anhui China; ^5^ North China University of Science and Technology Affiliated Hospital Tangshan China; ^6^ North China University of Science and Technology Tangshan Hebei China; ^7^ Hebei Key Laboratory of Medical Engineering and Integrated Utilization of Saline alkali Land Tangshan Hebei China; ^8^ Hebei Administration of TCM Key Laboratory of Quality Control of Salt alkali Resistant TCM Tangshan Hebei China; ^9^ School of Public Health North China University of Science and Technology Tangshan Hebei China; ^10^ The Center for Scientific Research The First Affiliated Hospital of Anhui Medical University Hefei China

**Keywords:** Bdkrb1, chemotaxis, m7G, Mettl1, neutrophil, psoriasis, RNA modifications

## Abstract

The functional significance of RNA modifications, specifically N7‐methylguanosine (m7G), in inflammatory conditions such as psoriasis remains not fully elucidated. This study demonstrates that methyltransferase‐like 1 (METTL1), an m7G methyltransferase, is significantly upregulated in epidermal keratinocytes of human psoriatic lesions and imiquimod (IMQ)‐induced murine models. Utilizing mice with an inducible keratinocyte‐specific *Mettl1* deletion (*Mettl1* ^fl/fl^ *Krt14*‐Cre^ERT2^), the research reveals significantly attenuated psoriasiform inflammation and decreased neutrophil infiltration relative to *Mettl1* ^fl/fl^ counterparts. Mechanistically, METTL1 drives inflammation by augmenting *Bdkrb1* mRNA stability through m7G modification. This stabilization leads to elevated bradykinin receptor B1 (BDKRB1) protein expression, which activates the p38 mitogen‐activated protein kinase (MAPK) pathway in keratinocytes, promoting the secretion of key proinflammatory C‐X‐C motif chemokine ligand (CXCL) chemokines and robust neutrophil chemotaxis. Crucially, both in vivo genetic BDKRB1 overexpression and pharmacological BDKRB1 activation successfully rescue the attenuated inflammatory phenotype in *Mettl1*‐deficient mice, firmly validating this specific signaling cascade. Conversely, pharmacological inhibition of the METTL1–BDKRB1 axis effectively mitigates psoriasiform inflammation. Collectively, these data establish that METTL1 modulates psoriasis by fostering p38‐dependent chemokine production and neutrophil recruitment, identifying the METTL1–BDKRB1 axis as a novel therapeutic target.

## Graphical abstract

This study unveils an unrecognized pro‐inflammatory epitranscriptomic checkpoint in psoriasis. By installing m7G modifications on the 5′ UTR of Bdkrb1 mRNA, METTL1 enhances receptor stability to orchestrate keratinocyte‐driven neutrophil recruitment via p38 MAPK signaling. Targeting this pathological cascade through combined METTL1 and BDKRB1 inhibition represents a synergistic and efficacious therapeutic approach.

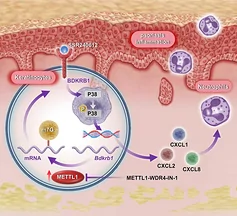

## 1 Introduction

Psoriasis is a chronic inflammatory skin disorder that affects a large proportion of the global population. Its pathology is defined by epidermal hyperproliferation, atypical keratinocyte differentiation, and pronounced immune cell infiltration [1, 2]. The pathogenesis of psoriasis is complex, driven by a self‐perpetuating cycle of inflammation involving innate and adaptive immunity [3]. A key axis in this process is the interleukin (IL)‐23/T helper cell (Th) 17 pathway [4]. IL‐23, which is secreted by dendritic cells, supports the proliferation and persistence of T cells that generate IL‐17 [5]. IL‐17A, in turn, targets keratinocytes directly, prompting them to release a variety of proinflammatory mediators (cytokines, chemokines, and antimicrobial peptides) that recruit more immune cells, thereby maintaining the inflammatory cycle [6].

Among the various immune cells recruited to psoriatic lesions, neutrophils play critical and multifaceted roles in the initiation and amplification of the inflammatory cascade [7, 8, 9]. Elevated numbers of neutrophils are found in psoriatic lesions, forming Munro's microabscesses in the stratum corneum and correlating with disease severity [10]. These neutrophils are an important source of reactive oxygen species, proteases, and proinflammatory mediators such as IL‐1β and tumor necrosis factor‐α (TNF‐α) [7, 11]. Keratinocyte‐derived chemokines, such as C‐X‐C motif chemokine ligand (CXCL) 1, CXCL2, and IL‐8 (CXCL8), are pivotal for this massive neutrophil recruitment [12]. The importance of this chemokine axis is underscored by its role in other inflammatory diseases, such as chronic obstructive pulmonary disease, acute respiratory distress syndrome, and rheumatoid arthritis [13, 14, 15]. Although the role of these chemokines as downstream effectors in psoriasis is well established, upstream regulatory mechanisms in keratinocytes remain less clear. Evidence from murine models indicates that neutrophil clearance can mitigate psoriasiform inflammation, but treatments specifically targeting these cells are not yet available for clinical use [16]. Despite their short lifespan, neutrophils are understood to create a pathogenic positive feedback mechanism in the chronic stage of the disease [4, 17].

The field of epitranscriptomics has recently highlighted post‐transcriptional RNA modifications as essential regulators of gene expression and the crosstalk between immune cells and epidermal keratinocytes that occurs in psoriasis [18, 19]. N6‐methyladenosine (m6A), the most common internal modification, has been studied widely in autoimmune contexts, including that of rheumatoid arthritis [20, 21, 22]. It is known to alter the stability and translation of key inflammatory transcripts in keratinocytes and immune cells, thereby shaping the progression of diseases such as psoriasis [23, 24]. The role of the m6A modification in psoriasis is cell type specific; the m6A level is decreased in psoriatic keratinocytes, inhibiting neutrophil chemotaxis by affecting the messenger (m)RNA stability of the lipid metabolism enzyme elongation of very long chain fatty acids 6 [23], but increased in psoriatic macrophages, enhancing interferon regulatory factor 5 signaling via solute carrier family 15 member 3 mRNA [24].

Another important, but less well‐characterized, modification is N7‐methylguanosine (m7G), catalyzed by the methyltransferase‐like 1 (METTL1)–WD repeat domain 4 (WDR4) complex [25, 26]. Classically, the m7G modification is known for its ubiquitous 5' cap mRNA structure (e.g., m7GpppX), which is essential for RNA integrity, protecting it from exonuclease degradation and ensuring its stability [27]. Although m7G is known to be critical for transfer RNA stability, accurate decoding, and the translation efficiency of specific mRNAs in the contexts of embryonic development and cancer [28, 29, 30], its function in inflammatory diseases remains largely unexplored [31].

In this study, we demonstrated that METTL1 expression is elevated in psoriatic skin. We identified *Bdkrb1* as a key post‐transcriptional target that METTL1 stabilizes via m7G modification. The resulting increase in bradykinin receptor B1 (BDKRB1) protein expression triggers p38 mitogen‐activated protein kinase (MAPK) signaling, promoting the secretion of chemokines that attract neutrophils. We propose that the therapeutic targeting of the METTL1–BDKRB1 pathway can dampen this inflammatory cascade, offering a new approach for psoriasis treatment.

## 2 Results

### 2.1 The METTL1–WDR4 Complex and m7G Modification are Concurrently Upregulated in Psoriatic Lesions and Experimental Psoriasis Models

**FIGURE 1:**
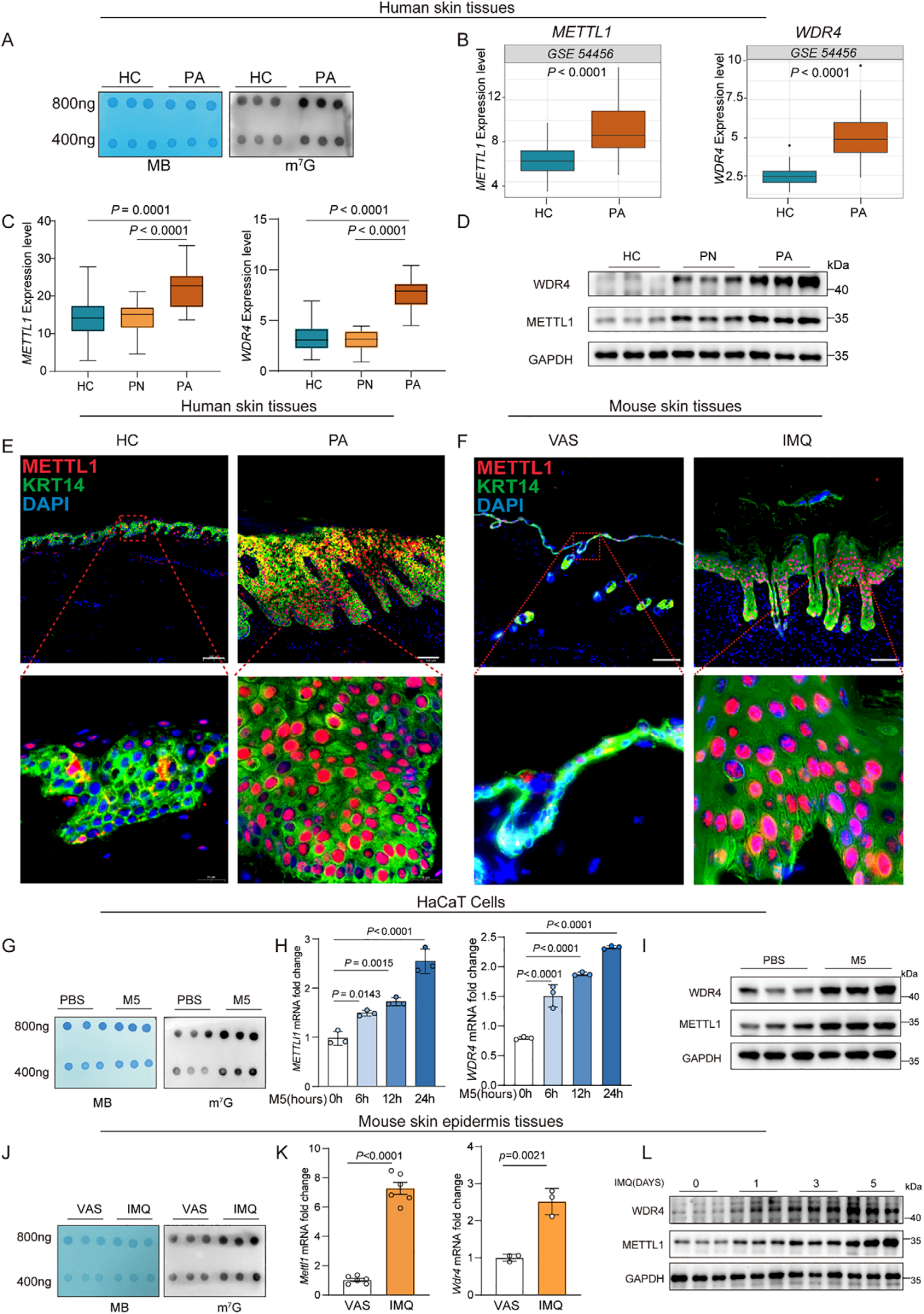
The METTL1–WDR4 complex and m7G modification are concurrently upregulated in psoriatic lesions and experimental psoriasis models. (A) m7G abundance in healthy control (HC) and psoriatic lesional skin (PA), determined by dot blot analysis. Methylene blue (MB) was used as the loading control. (*n* = 3 per group). (B) *METTL1* and *WDR4* mRNA expression levels from the GSE54456 dataset (HC vs. PA). (C) *METTL1* and *WDR4* mRNA expression in human HC(*n* = 20), psoriasis non‐lesional (PN) (*n* = 21), and PA skin tissues(*n* = 21). (D) Western blot analysis of WDR4 and METTL1 protein expression in human HC, PN, and PA skin tissues. GAPDH was used as the loading control. (*n* = 3 per group). (E) Representative immunofluorescence images of KRT14 (green; keratinocytes), METTL1 (red), and DAPI (blue; nuclei) staining in human HC and PA skin sections. Red dashed boxes indicate the magnified areas. Scale bars = 100 µm (top) and 20 µm (bottom). (F) Representative immunofluorescence images of KRT14 (green), METTL1 (red), and DAPI (blue) in dorsal skin from mice treated topically with Vaseline (VAS) or imiquimod (IMQ). Scale bars = 100 µm. (G) m7G abundance in HaCaT cells treated with phosphate‐buffered saline (PBS) or M5 cytokine cocktail, determined by dot blot analysis. (*n* = 3 per group). (H) *METTL1* and *WDR4* mRNA expression in HaCaT cells treated with M5 cytokine cocktail for 0, 6, 12, and 24 h (*n* = 3 per group), determined by real‐time PCR. (I) Western blot analysis of WDR4 and METTL1 protein expression in HaCaT cells treated with PBS or M5 cytokine cocktail. (*n* = 3 per group). (J) m7G abundance in VAS‐ and IMQ‐treated mouse epidermis, determined by dot blot analysis. (K) *Mettl1* and *Wdr4* mRNA expression in VAS‐ and IMQ‐treated mouse epidermis (*n* = 3 per group), determined by real‐time PCR. (L) Western blot analysis of WDR4 and METTL1 protein expression in wild‐type mice treated topically with IMQ for 0, 1, 3, and 5 consecutive days. (*n* = 3). Data are representative of three independent experiments and are shown as mean ± SD. Statistical significance was determined by unpaired Student's *t*‐test (B, K) or one‐way analysis of variance with Tukey's *post hoc* test (C, H). ns, not significant.

Using an m7G dot blot analysis, we found a greater abundance of the m7G modification in RNA from human psoriatic lesional skin (PA) compared to healthy control skin (HC) (Figure 1A). Because N7‐methylguanosine modification is primarily catalyzed by the METTL1–WDR4 complex, we assessed their expression profiles. Analysis of the public RNA‐seq dataset (GSE54456) and our internal clinical cohort revealed that the mRNA levels of both *METTL1* and *WDR4* were significantly higher in psoriatic lesions (PA) than in paired non‐lesional (PN) and healthy control skin (HC) (Figure 1B,C). This significant upregulation was further confirmed by qPCR analysis of peripheral blood cells from psoriasis patients (Figure S1A) and Western blotting of skin tissue lysates (Figure 1D). Consistently, dual immunofluorescence (IF) and immunohistochemistry (IHC) staining confirmed the precise colocalization of METTL1 with the keratinocyte marker KRT14, highlighting a marked accumulation of METTL1‐positive keratinocytes in psoriatic tissues compared to healthy controls (Figure 1E and Figure S1B).

Given that both m7G abundance and the METTL1–WDR4 complex are coordinately upregulated in epidermal keratinocytes within psoriatic lesions, we utilized the HaCaT cell line treated with a well‐validated proinflammatory cytokine cocktail (M5: IL‐17A, TNF‐α, IL‐1α, IL‐22, and oncostatin M) to recapitulate the psoriatic inflammatory environment in vitro. M5 stimulation led to a robust, time‐dependent increase in *METTL1* and *WDR4* mRNA and protein expression, which directly corresponded to an elevated total m7G level (Figure 1G–I).

In vivo, utilizing the classic imiquimod (IMQ)‐induced psoriasis‐like model, the abundance of METTL1‐positive keratinocytes in the epidermis was markedly increased relative to the Vaseline (VAS) control group (Figure 1F). This epithelial upregulation correlated with a significantly greater abundance of the m7G modification in epidermal RNA from IMQ‐treated mice (Figure 1J). During the progression of the IMQ model, both *Mettl1* and *Wdr4* mRNA levels, alongside their corresponding protein expression, were dynamically elevated, peaking during the most intense inflammatory phase (Figure 1K,L). Notably, METTL1 protein expression remained significantly elevated above baseline levels even 30 days after the cessation of IMQ treatment, suggesting a sustained epidermal priming effect (Figure S1C). To further corroborate these findings, we employed supplementary cytokine‐induced murine models. Consistently, a prominent accumulation of METTL1‐positive epidermal cells was also observed in both IL‐17A‐ and IL‐23A‐induced psoriasis‐like mice compared to PBS‐treated controls (Figure S1D). Together, these consistent clinical, in vitro, and in vivo data strongly indicate that the m7G methyltransferase complex is hyperactivated during psoriatic inflammation.

### 2.2 Epidermal METTL1 Deficiency Attenuates Psoriatic Inflammation

As our clinical and in vivo data (Figure 1) demonstrated that the upregulation of the m7G modification predominantly occurs within epidermal keratinocytes, we first sought to determine its cell‐intrinsic function in vitro. Although both components of the METTL1–WDR4 complex are elevated during psoriatic inflammation, METTL1 functions as the indispensable catalytic core responsible for m7G addition, whereas WDR4 serves primarily as a structural adaptor. Therefore, we specifically targeted METTL1 to directly abolish the complex's methyltransferase activity. Following M5 cytokine cocktail stimulation, *METTL1*‐knockdown (sh*METTL1*) HaCaT cells displayed significantly lower proinflammatory gene expression compared with control (shNC) cells (Figure S2A). Beyond its effect on inflammatory gene expression, *METTL1* knockdown also altered keratinocyte proliferative and differentiation behavior under inflammatory conditions. Notably, in the absence of M5 stimulation, *METTL1*‐deficient HaCaT cells showed no significant differences in proliferation or differentiation markers compared with controls; however, upon M5 stimulation, cell cycle analysis revealed a significant increase in the G0/G1 phase population and a concomitant reduction in S phase entry (Figure S2B). Similarly, in a calcium‐induced differentiation model, *METTL1* knockdown reduced *KI67* expression and upregulated the terminal differentiation markers *KRT10*, *LOR*, and *FLG* only under M5 stimulation at both 48 and 72 h (Figure S2C).

**FIGURE 2:**
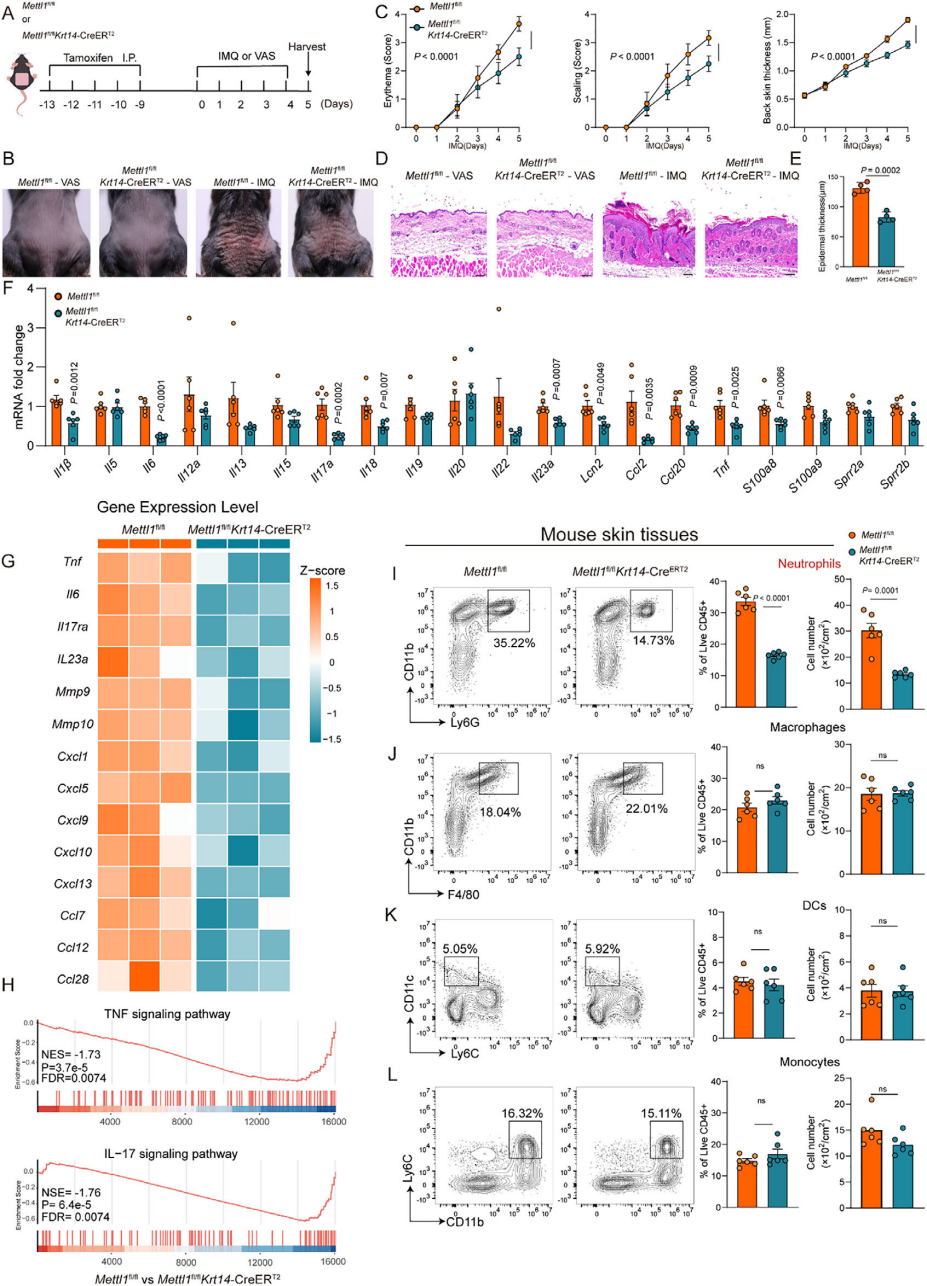
Epidermal METTL1 Deficiency Attenuates Psoriatic Inflammation. (A) Schematic representation of the tamoxifen induction and IMQ treatment protocol. (B) Representative photographs of mouse back skin. (C) Clinical scoring of erythema, scaling, and back skin thickness for mice treated with IMQ for 5 consecutive days. (*n* = 6 per group). (D) Representative hematoxylin and eosin (H&E) staining of skin sections. Scale bars = 100 µm. (E) Epidermal thickness, measured from H&E‐stained images. (*n* = 6 per group). (F) mRNA fold change of pathogenic factors in IMQ‐treated mouse skin, determined by qPCR (*n* = 6 per group). (G) Heatmap showing the expression (Z‐score) of selected genes involved in immune responses in *Mettl1*fl/fl and *Mettl1* ^fl/fl^ *Krt14*‐Cre^ERT2^ mouse skin, obtained by RNA‐seq. (*n* = 3 per group). (H) Gene set enrichment analysis (GSEA) plots for the TNF and IL‐17 signaling pathways comparing *Mettl1* ^fl/fl^ and *Mettl1* ^fl/fl^ *Krt14*‐Cre^ERT2^ mouse skin, obtained by RNA‐seq. (I–L) Representative flow cytometry plots and quantifications (percentage of live CD45+ cells and cell number per cm^2^) of (I) neutrophils, (J) macrophages, (K) DCs, and (L) monocytes in mouse skin tissues (*n* = 6 per group). Data are representative of three independent experiments and are shown as mean ± SD. Statistical significance was determined by unpaired Student's *t*‐test (C, E, F, I–L). ns, not significant.

To evaluate the in vivo contribution of this epidermal m7G modification, we generated an inducible keratinocyte‐specific *Mettl1*‐deficient mouse model by crossing *Mettl1* ^fl/fl^ mice with *Krt14*‐Cre^ERT2^ mice (Figure 2A and Figure S3A). Macroscopic evaluation and clinical scoring demonstrated that epidermal *Mettl1* deficiency significantly attenuated the severity of imiquimod (IMQ)‐induced psoriasis‐like manifestations, reflecting decreased back skin thickness, reduced erythema, and alleviated scaling compared with IMQ‐treated *Mettl1* ^fl/fl^ control mice (Figure 2B,C). Histological analysis of skin sections further confirmed a marked reduction in epidermal thickness in the *Mettl1*‐deficient group (Figure 2D,E). Consistent with the in vitro observations, qPCR analysis of IMQ‐treated *Mettl1*fl/fl*Krt14*‐CreERT2 epidermis revealed significantly decreased *Mki67* expression and concurrently elevated mRNA levels of *Krt10*, *Lor*, and *Flg* compared with *Mettl1*fl/fl controls (Figure S3B). Immunohistochemical staining further corroborated these findings at the protein level, showing diminished KI67‐positive proliferating cells and enhanced expression of K10, Loricrin, and Filaggrin in the *Mettl1*‐deficient epidermis (Figure S3C).

RNA‐seq analysis of whole skin samples revealed a significant downregulation of psoriasis‐associated pathogenic genes, including those encoding crucial cytokines (e.g., *Tnf*, *Il17ra*), matrix metalloproteinases (e.g., *Mmp9*, *Mmp10*), and chemokines (e.g., *Cxcl1*, *Cxcl5*), in *Mettl1* ^fl/fl^ *Krt14*‐Cre^ERT2^ mice relative to *Mettl1* ^fl/fl^ mice (Figure 2G). These findings were supported by quantitative PCR (qPCR) validation (Figure 2F). Moreover, gene set enrichment analysis (GSEA) demonstrated that gene sets related to the IL‐17 and TNF signaling pathways, which represent the fundamental pathogenic axes in psoriasis [32, 33], were significantly suppressed in the *Mettl1* ^fl/fl^ *Krt14*‐Cre^ERT2^ group (Figure 2H).

Then we investigated the immune cell landscape in the skin lesions. Flow cytometry analysis showed a significant reduction in both the percentage and absolute number of infiltrating neutrophils in *Mettl1* ^fl/fl^ *Krt14*‐Cre^ERT2^ skin compared with *Mettl1* ^fl/fl^ control skin (Figure 2I and Figure S4A,B). In contrast, no significant differences in the proportions of macrophages, dendritic cells (DCs), or monocytes were observed between the groups (Figure 2J–L). Additionally, flow cytometric analysis revealed that neither the proportion nor the absolute number of CD4^+^ or CD8^+^ T cells, nor the CD4^+^/CD8^+^ ratio, was significantly altered in *Mettl1*fl/fl*Krt14*‐CreERT2 mice compared with controls (Figure S4C,D).

### 2.3 Epidermal METTL1 Orchestrates Neutrophil Recruitment via the CXCL Chemokine Axis

**FIGURE 3:**
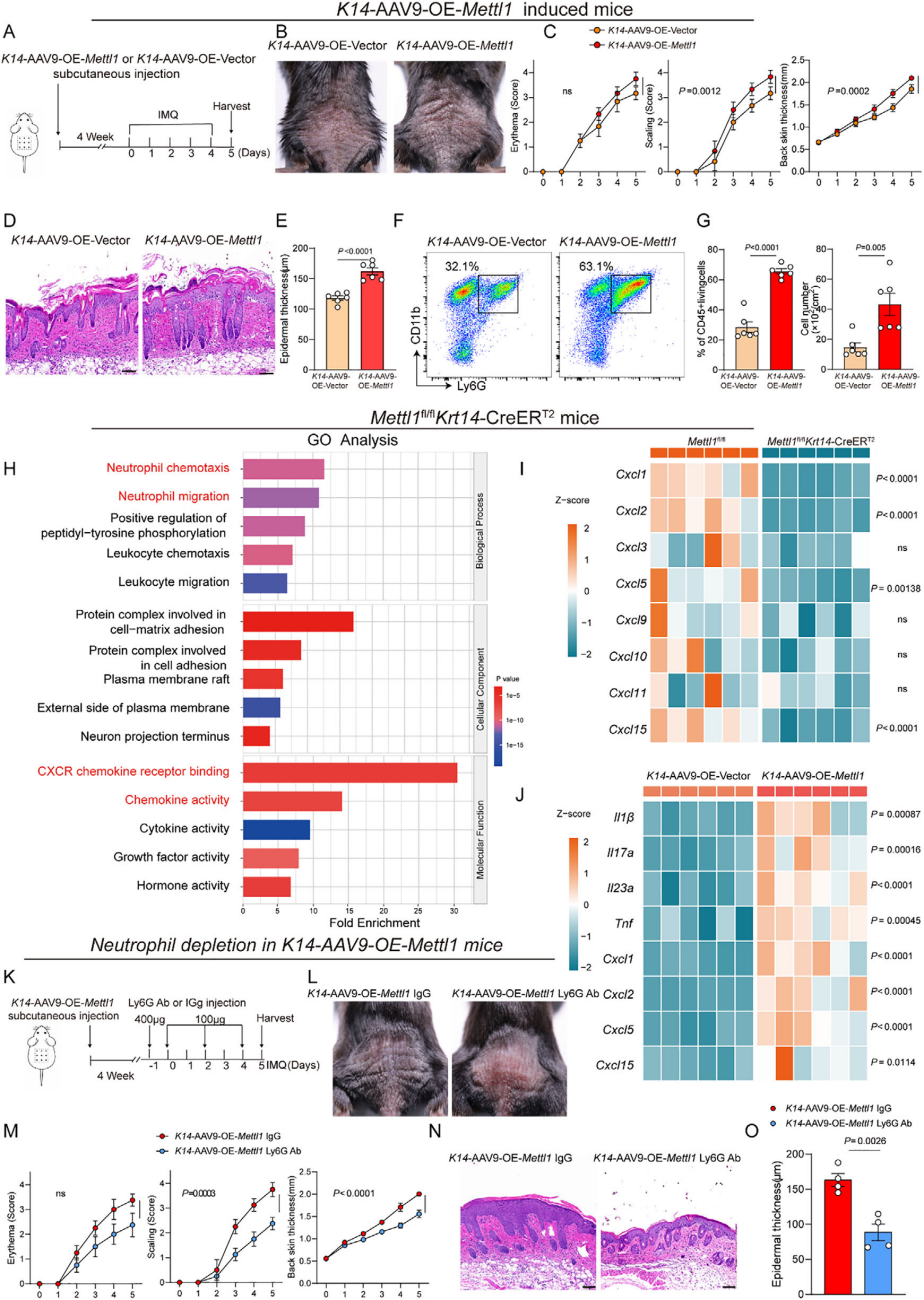
Epidermal METTL1 drives psoriatic inflammation by promoting CXCL‐mediated neutrophil chemokine axis. (A) Schematic representation of the K14‐AAV9‐Vector and K14‐AAV9‐OE‐*Mettl1* mouse models. (B) Representative photographs of mouse back skin. (C) Clinical scoring of erythema, scaling, and back skin thickness for mice treated with IMQ for 5 consecutive days (*n* = 6 per group). (D) Representative hematoxylin and eosin (H&E) staining of skin sections. Scale bars = 100 µm. (E) Epidermal thickness, measured from H&E‐stained images. (*n* = 6 per group). (F, G) Representative flow cytometry plots (F) and quantification (G) of infiltrating neutrophils in K14‐AAV9‐Vector and K14‐AAV9‐OE‐*Mettl1* mice after 5 days of IMQ treatment. (*n* = 6 per group). (H) Gene Ontology (GO) terms enriched in differentially expressed genes between *Mettl1*fl/fl and *Mettl1*fl/fl*Krt14*‐CreERT2 mouse skin, identified by RNA‐seq. (I) Heatmap of *Cxcl* chemokine mRNA expression (Z‐score) in *Mettl1*fl/fl and *Mettl1*fl/fl*Krt14*‐CreERT2 mouse epidermis after IMQ treatment (*n* = 6 per group), determined by qPCR. (J) Heatmap showing the expression of proinflammatory cytokines and *Cxcl* chemokines in K14‐AAV9‐Vector and K14‐AAV9‐OE‐*Mettl1* mouse epidermis after IMQ treatment (*n* = 6 per group), determined by qPCR. (K) Schematic representation of the neutrophil depletion experiment using an anti‐Ly6G antibody in K14‐AAV9‐OE‐*Mettl1* mice. (L) Representative photographs of mouse back skin. (M) Clinical scoring of erythema, scaling, and back skin thickness (*n* = 4 per group). (N) Representative H&E staining of skin sections. Scale bars = 100 µm. (O) Epidermal thickness, measured from H&E‐stained images (*n* = 4 per group). Data are representative of three independent experiments and are shown as mean ± SD. Statistical significance was determined by unpaired Student's *t*‐test (C, E, G, I, J, M, O). ns, not significant.

To investigate the in vivo sufficiency of METTL1 in driving the disease, we employed an adeno‐associated virus 9 vector to achieve keratinocyte‐specific *Mettl1* overexpression (K14‐AAV9‐OE‐*Mettl1*), utilizing an empty vector (K14‐AAV9‐Vector) as a control (Figure 3A). Macroscopic evaluation and clinical scoring demonstrated that epidermal *Mettl1* overexpression exacerbated the severity of IMQ‐induced psoriasis‐like manifestations, reflecting increased back skin thickness, aggravated erythema, and severe scaling compared with vector‐treated controls (Figure 3B,C). This exacerbated phenotype was accompanied by increased epidermal thickness (Figure 3D,E) and an accumulation of infiltrating neutrophils (Figure 3F,G).

Corroborating the flow cytometry data, Gene Ontology (GO) analysis of RNA‐seq data from IMQ‐treated skin revealed that biological processes associated with “neutrophil migration” and “neutrophil chemotaxis” were highly enriched in *Mettl1* ^fl/fl^ mice compared with *Mettl1* ^fl/fl^ *Krt14*‐Cre^ERT2^ mice (Figure 3H). The GO analysis also highlighted an enrichment in molecular functions such as “CXCR chemokine receptor binding” and “chemokine activity.” Because CXCL1, CXCL2, and CXCL8 are well‐documented as the primary keratinocyte‐derived chemokines responsible for recruiting neutrophils into psoriatic lesions via CXCR1/2 receptors [12, 34], we hypothesized that METTL1 directly regulates this specific epidermal chemokine network.

Immunofluorescence (IF) analysis visually confirmed a notable accumulation of Ly6G‐positive neutrophils near the dermal–epidermal junction in K14‐AAV9‐OE‐*Mettl1* mice, whereas *Mettl1* deficiency markedly decreased this infiltration (Figure S5A,B). At the molecular level, *METTL1* knockdown in human HaCaT cells led to a reduction in the expression of key neutrophil‐attracting chemokines, notably *CXCL1*, *CXCL2*, and *CXCL8* at both the mRNA (Figure S5C) and secreted protein (Figure S5D) levels. Because the murine genome lacks a direct true ortholog for human *CXCL8*, mice rely on functional homologs—including Cxcl1, Cxcl2, Cxcl5, and Cxcl15—which act as CXCR2 ligands to drive neutrophil chemotaxis [35]. Correspondingly, IMQ‐treated *Mettl1* ^fl/fl^ *Krt14*‐Cre^ERT2^ mouse skin exhibited significantly reduced mRNA expression of these functional orthologs (Figure 3I), and ELISA of serum confirmed a concordant decrease in CXCL1, CXCL2, CXCL5, and CXCL15 protein levels (Figure S5E). Immunohistochemical staining further corroborated diminished CXCL1 expression in the *Mettl1*‐deficient epidermis (Figure S5F). Conversely, *Mettl1* overexpression upregulated these CXCR2‐targeting chemokines along with other classical proinflammatory cytokines (Figure 3J), with ELISA analysis confirming elevated secreted protein levels of CXCL1, CXCL2, CXCL5, and CXCL15 (Figure S5G).

To determine whether the exacerbated psoriasis‐like inflammation in *Mettl1*‐overexpressing mice functionally relies on this neutrophil recruitment, we performed an in vivo neutrophil depletion assay by administering an anti‐Ly6G neutralizing antibody, using isotype IgG‐treated mice as a control (Figure 3K and Figure S5H). The targeted depletion of neutrophils substantially mitigated the severe skin inflammation induced by *Mettl1* overexpression, reducing back skin thickness, erythema, scaling, and epidermal hyperplasia (Figure 3L–O).

### 2.4 Integrated Epitranscriptomic Profiling Identifies *Bdkrb1* as the Conserved Target of METTL1

**FIGURE 4:**
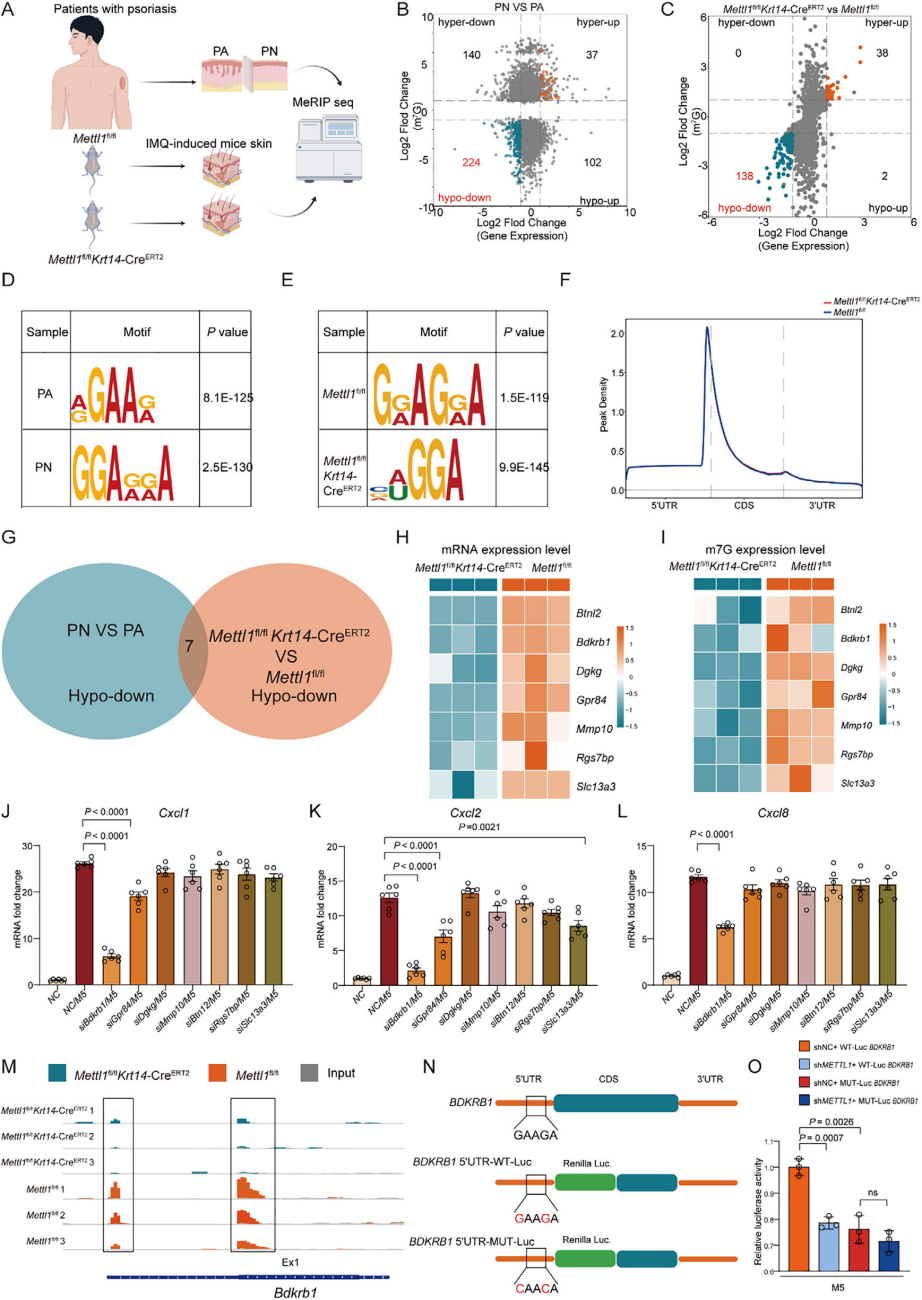
Integrated Epitranscriptomic Profiling Identifies *Bdkrb1* as the Conserved Target of METTL1. (A) Schematic illustration of sample collection and the MeRIP‐seq/RNA‐seq workflow. (B, C) Scatter plots showing the correlation between log2 fold changes in m7G peak intensity and global gene expression in (B) human non‐lesional (PN) vs. psoriatic lesional (PA) skin, and (C) *Mettl1* ^fl/fl^ vs. *Mettl1* ^fl/fl^ *Krt14*‐Cre^ERT2^ mouse skin. (D, E) Conserved sequence motifs highly enriched within the m7G peaks identified in (D) human and (E) mouse datasets. (F) Density distribution of m7G peaks across methylated mRNA transcripts, indicating enrichment near the 5′ UTR and start codon. (G) Venn diagram illustrating the intersection of hypomethylated and downregulated (hypo‐down) genes between the human and mouse datasets, yielding 7 conserved candidates. (H, I) Heatmaps showing the (H) mRNA expression levels and (I) m7G peak intensities of the 7 candidate genes in *Mettl1*fl/fl and *Mettl1*fl/fl*Krt14*‐CreERT2 mouse skin. (J–L) mRNA fold change of human neutrophil chemokines (J) *CXCL1*, (K) *CXCL2*, and (L) *CXCL8* in HaCaT cells following siRNA‐mediated knockdown of the individual candidate genes under M5 stimulation (*n* = 6 per group), determined by qPCR. (M) Visualization of MeRIP‐seq tracks (IGV) showing the abundance of m7G peaks across the *Bdkrb1* locus in *Mettl1*fl/fl and *Mettl1*fl/fl*Krt14*‐CreERT2 epidermis. The black box highlights the diminished m7G peak at the 5′ UTR region. (N) Schematic representation of the dual‐luciferase reporter constructs harboring either the wild‐type (WT‐Luc, containing the GAAGA motif) or mutated (MUT‐Luc, CAACA motif) *BDKRB1* 5′ UTR sequences upstream of the Renilla luciferase gene. (O) Relative luciferase activity of the WT and MUT *BDKRB1* reporters in control (NC) or *METTL1*‐knockdown (KD) HaCaT cells stimulated with the M5 cocktail (*n* = 3). Data are representative of three independent experiments and are shown as mean ± SD. Statistical significance was determined by one‐way analysis of variance with Tukey's *post hoc* test (J–L, O). ns, not significant.

To elucidate the specific mRNA targets responsible for the METTL1‐driven inflammatory cascade, we performed parallel m7G methylated RNA immunoprecipitation sequencing (MeRIP‐seq) and RNA‐seq (Figure 4A). Scatter plot analysis revealed a positive correlation between the m7G modification levels and global mRNA expression in both human psoriatic tissues (PA vs. PN) and the IMQ‐induced murine model (*Mettl1* ^fl/fl^ *Krt14*‐Cre^ERT2^ vs. *Mettl1* ^fl/fl^). Specifically, *Mettl1* deficiency led to a reduction in m7G peaks paired with significant target gene downregulation (hypo‐down), indicating that m7G methylation enhances mRNA transcript stability (Figure 4B,C). Motif analysis across both human and mouse datasets identified two conserved GA‐rich consensus motifs characteristic of m7G modification (Figure 4D,E). Furthermore, these METTL1‐dependent m7G peaks were predominantly enriched within the 5′ untranslated regions (5′ UTRs) adjacent to the start codons (Figure 4F).

Given that single‐species high‐throughput sequencing often yields context‐dependent alterations, cross‐species transcriptomic integration serves as a robust strategy to filter out species‐specific noise and prioritize evolutionarily conserved pathogenic drivers [36]. Therefore, we intersected the human and murine “hypo‐down” gene sets, yielding an intersection of seven conserved candidate genes (*Btnl2*, *Bdkrb1*, *Dgkg*, *Gpr84*, *Mmp10*, *Rgs7bp*, and *Slc13a3*) (Figure 4G–I). Quantitative PCR (qPCR) validation confirmed that epidermal *Mettl1* ablation led to the significant downregulation of these candidates in the murine model (Figure S5I). We next performed a functional screening by individually knocking down these genes in human HaCaT cells. Only the silencing of *BDKRB1* concurrently attenuated the M5‐induced upregulation of the key neutrophil‐attracting chemokines *CXCL1*, *CXCL2*, and *CXCL8* (Figure 4J–L).

Direct examination of the MeRIP‐seq tracks visualized a METTL1‐dependent m7G peak specifically localized within the 5′ UTR of the *Bdkrb1* transcript, which was markedly diminished upon *Mettl1* ablation (Figure 4M). To verify the functional dependence of *Bdkrb1* stability on this 5′ UTR methylation site, we engineered dual‐luciferase reporter constructs containing either the wild‐type (WT‐Luc, harboring the GAAGA motif) or a specifically mutated (MUT‐Luc, converted to CAACA) *BDKRB1* 5′ UTR sequence (Figure 4N). Under M5 inflammatory stimulation, *METTL1* knockdown in HaCaT cells significantly impaired the luciferase activity driven by the WT *BDKRB1* reporter. Conversely, the activity of the MUT reporter remained largely unaffected by METTL1 status (Figure 4O). These data identify the *Bdkrb1* transcript as a functional target of METTL1, relying on 5′ UTR m7G methylation to maintain its stability.

To further validate that the GAAGA motif within the *BDKRB1* 5′ UTR is the bona fide cis‐element responsible for METTL1‐mediated regulation, we performed site‐directed mutagenesis on the full‐length *BDKRB1* construct, converting the same 5′ UTR GAAGA motif used in the luciferase reporter (Figure 4N) to CAACA, and reintroduced either the wild‐type (WT) or 5′ UTR‐mutated (MUT) *BDKRB1* into HaCaT cells together with sh NC or sh *METTL1* shRNA. Actinomycin D chase assays showed that *BDKRB1* transcript stability was markedly reduced upon either *METTL1* knockdown or 5′ UTR mutation (Figure S6A). Consistently, BDKRB1 protein expression was diminished accordingly (Figure S6B), and the induction of the key neutrophil‐attracting chemokines CXCL1, CXCL2, and CXCL8 at both the mRNA (Figure S6C) and secreted protein (Figure S6D) levels was correspondingly suppressed. Collectively, these site‐directed mutagenesis data establish a strict requirement for the 5′ UTR GAAGA m7G motif within *BDKRB1* in mediating METTL1‐driven *Bdkrb1* stabilization and the downstream chemokine response.

### 2.5 METTL1‐Stabilized *Bdkrb1* Activates the p38 MAPK Pathway to Drive Chemokine Production

**FIGURE 5:**
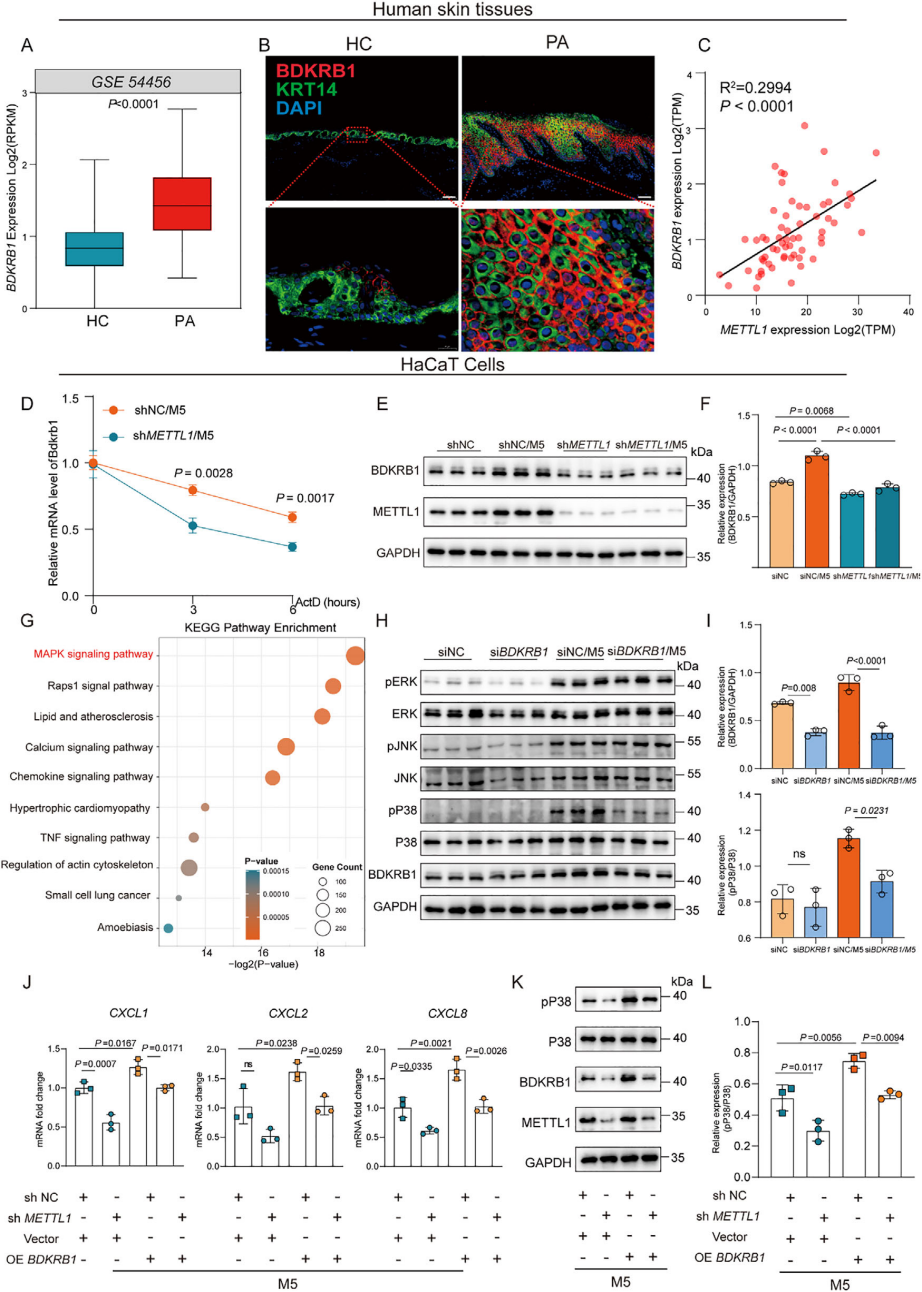
METTL1 stabilizes *Bdkrb1* mRNA to promote p38‐mediated chemokine production. (A) *BDKRB1* mRNA expression (Log2 RPKM) from the GSE54456 dataset (HC vs. PA). (B) Representative dual immunofluorescence images of BDKRB1 (red), KRT14 (green), and DAPI (blue) in human healthy control (HC) and psoriatic lesional (PA) skin sections. Scale bars = 100 µm (top) and 20 µm (bottom). (C) The relationships between *METTL1* and *BDKRB1* mRNA expression levels (Log2 TPM) in human skin tissues. (*n* = 62). The goodness of fit (R^2^) and statistical significance (*p* value) are indicated within the graph. (D) *BDKRB1* mRNA decay rate in control (shNC) and *METTL1*‐knockdown (sh*METTL1*) HaCaT cells following actinomycin D (ActD) treatment. (*n* = 3 per group). (E, F) Western blot analysis (E) and quantification (F) of BDKRB1 and METTL1 protein expression in HaCaT cells with or without M5 stimulation. (*n* = 3 per group). (G) KEGG pathway enrichment analysis highlighting the MAPK signaling pathway. (H, I) Western blot analysis (H) and quantification (I) of phosphorylated and total ERK, JNK, and p38, alongside BDKRB1, in control (siNC) and *BDKRB1*‐knockdown (si*BDKRB1*) HaCaT cells. (*n* = 3 per group). (J) mRNA fold change of *CXCL1*, *CXCL2*, and *CXCL8* in HaCaT cells across different rescue conditions (shNC, sh*METTL1*, Vector, OE *BDKRB1*) under M5 stimulation (*n* = 3 per group), determined by qPCR. (K, L) Western blot analysis (K) and quantification (L) of pP38, P38, B1R (BDKRB1), and METTL1 in the indicated rescue groups under M5 stimulation. (*n* = 3 per group). Data are representative of three independent experiments and are shown as mean ± SD. Statistical significance was determined by unpaired Student's *t*‐test (A), linear regression analysis (C) or one‐way analysis of variance with Tukey's *post‐hoc* test (D, F, I, J, L). ns, not significant.

Consistent with its role as a downstream target of METTL1, *BDKRB1* mRNA expression was significantly elevated in human psoriatic lesions compared with healthy control skin, as evidenced by the public GSE54456 dataset (Figure 5A). Dual immunofluorescence (IF) analysis confirmed this upregulation, localizing the BDKRB1 protein predominantly to the epidermal keratinocytes in psoriatic patient samples (Figure 5B). Furthermore, this upregulation was corroborated in our internal clinical cohort (Figure S6E), where *BDKRB1* expression exhibited a positive correlation with *METTL1* expression (Figure 5C).

To establish that METTL1‐mediated m7G modification intrinsically regulates *Bdkrb1* expression, we first assessed transcript stability in vitro. Following transcriptional inhibition with actinomycin D, *METTL1* knockdown significantly shortened the half‐life of *BDKRB1* mRNA in human HaCaT cells (Figure 5D). This accelerated mRNA decay led to a corresponding decrease in BDKRB1 protein expression (Figure 5E,F). Next, we validated this regulatory effect in vivo. Consistent with the in vitro findings, epidermal *Mettl1* ablation reduced both *Bdkrb1* mRNA and protein levels in IMQ‐treated mice (Figure S6F–H). Conversely, targeted *Mettl1* overexpression elevated BDKRB1 expression in the skin lesions (Figure S6I–K).

To link BDKRB1 upregulation to chemokine secretion, we performed KEGG pathway enrichment analysis, which implicated the MAPK signaling pathway (Figure 5G). In M5‐stimulated HaCaT cells, targeted *BDKRB1* knockdown specifically impaired the phosphorylation of p38 MAPK, without exerting a discernible effect on the phosphorylation of ERK or c‐Jun N‐terminal kinase (JNK) (Figure 5H,I).

Finally, to validate the functional role of the METTL1–BDKRB1–p38 signaling axis, we conducted in vitro rescue assays. *METTL1* knockdown in HaCaT cells significantly attenuated the M5‐induced upregulation of key neutrophil chemokines and suppressed p38 phosphorylation. These suppressive effects were significantly rescued by the concomitant ectopic overexpression of *BDKRB1* at both the mRNA (Figure 5J) and secreted protein (Figure S6L) levels, along with restored p38 phosphorylation (Figure 5K,L). Collectively, these results demonstrate that METTL1 enhances *Bdkrb1* mRNA stability, which subsequently triggers p38 MAPK activation to drive chemokine production.

### 2.6 Pharmacological BDKRB1 Activation Significantly Rescues the Attenuated Psoriatic Phenotype in *Mettl1*‐Deficient Mice

**FIGURE 6:**
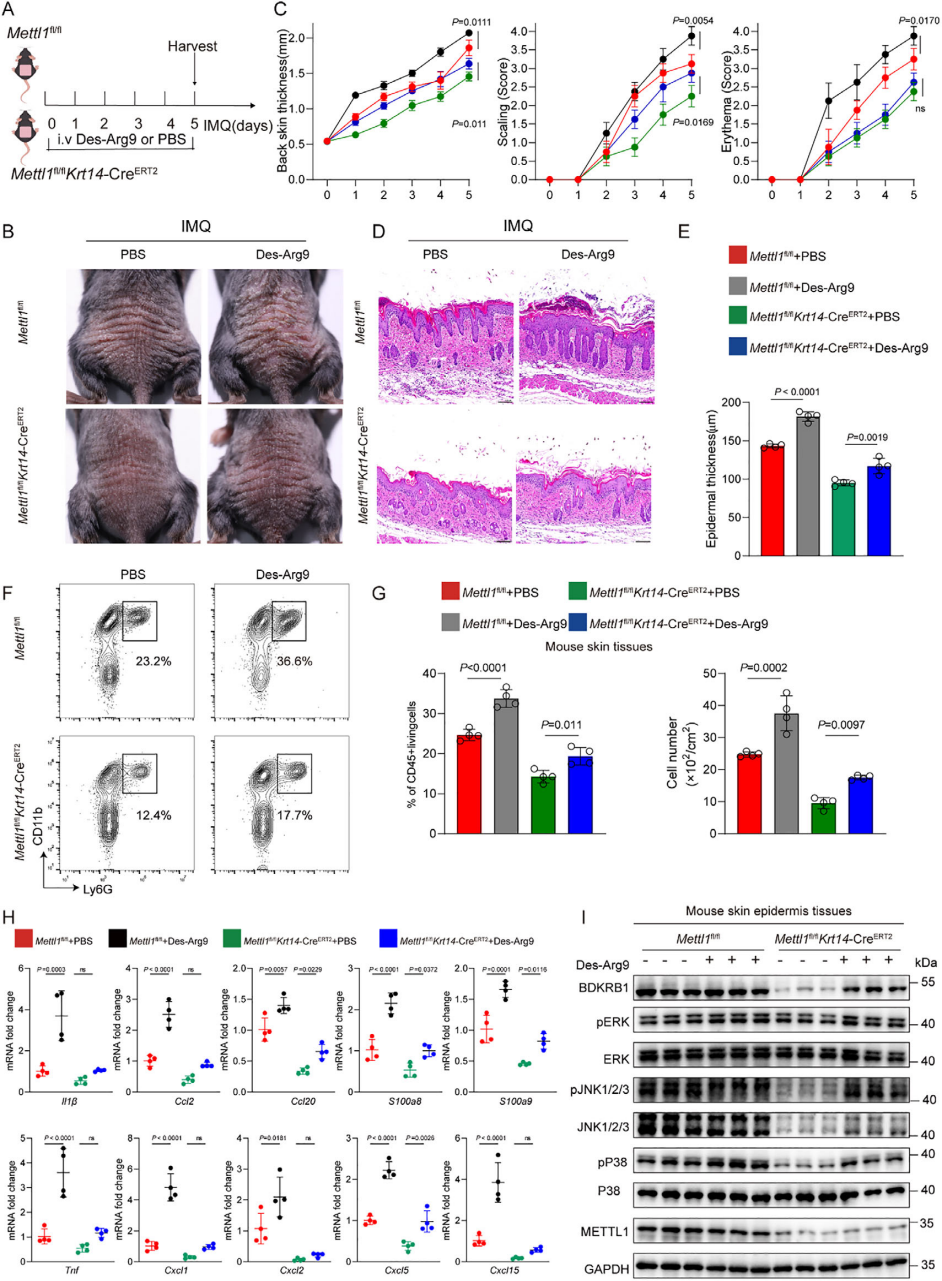
Pharmacological BDKRB1 Activation Significantly Rescues the Attenuated Psoriatic Phenotype in *Mettl1*‐Deficient Mice. (A) Schematic illustrating the in vivo rescue experiment using the BDKRB1 agonist (Des‐Arg9) via intravenous injection in *Mettl1* ^fl/fl^ and *Mettl1* ^fl/fl^ *Krt14*‐Cre^ERT2^ mice during IMQ treatment. (B) Representative macroscopic photographs of mouse back skin. (C) Clinical scoring of back skin thickness, scaling, and erythema for mice treated with IMQ for 5 consecutive days (*n* = 4 per group). (D) Representative hematoxylin and eosin (H&E) staining of skin sections. Scale bars = 100 µm. (E) Epidermal thickness, measured from H&E‐stained images. (*n* = 4 per group). (F, G) Representative flow cytometry plots (F) and statistical quantification (G) of the percentage and absolute number of infiltrating neutrophils in mouse skin tissues (*n* = 4 per group). (H) mRNA fold change of key proinflammatory cytokines and *Cxcl* chemokines in the mouse epidermis across the indicated treatment groups (*n* = 4 per group), determined by qPCR. (I) Western blot analysis of BDKRB1, phosphorylated and total ERK, JNK, and p38, alongside METTL1, in the mouse epidermis across the indicated treatment groups. (*n* = 4 per group). GAPDH was used as the loading control. Data are representative of three independent experiments and are shown as mean ± SD. Statistical significance was determined by one‐way analysis of variance with Tukey's *post hoc* test (C, E, G, H). ns, not significant.

To validate the in vivo functional dependence of psoriatic inflammation on the METTL1–BDKRB1–p38 axis, *Mettl1* ^fl/fl^ and *Mettl1* ^fl/fl^ *Krt14*‐Cre^ERT2^ mice were administered the BDKRB1 agonist, Des‐Arg9‐Bradykinin (Des‐Arg9), via intravenous injection during the progression of IMQ‐induced psoriasis (Figure 6A).

In IMQ‐treated *Mettl1* ^fl/fl^ mice, Des‐Arg9 treatment exacerbated the psoriasis‐like manifestations. BDKRB1 agonist administration to IMQ‐treated *Mettl1* ^fl/fl^ *Krt14*‐Cre^ERT2^ mice successfully, though partially, rescued the attenuated psoriatic phenotype. According to the operational model of pharmacological agonism, the maximal achievable response is fundamentally constrained by tissue receptor density [37]. Given that *Mettl1* deficiency inherently depletes the available pool of BDKRB1 receptors, exogenous pharmacological activation yielded a significant but submaximal phenotypic rescue, rather than a complete reversal. This intervention substantially restored macroscopic disease manifestations, reflecting increased back skin thickness, severe scaling, and aggravated erythema (Figure 6B,C). This clinical exacerbation was accompanied by significantly increased epidermal thickness at the histological level (Figure 6D,E).

At the molecular level, Des‐Arg9 treatment upregulated the epidermal expression of psoriasis‐associated proinflammatory cytokines and key neutrophil‐attracting *Cxcl* chemokines, shifting their expression toward the levels observed in *Mettl1* ^fl/fl^ mice (Figure 6H), and ELISA of serum confirmed a corresponding restoration of CXCL1, CXCL2, CXCL5, and CXCL15 protein levels (Figure S7A). Consequently, flow cytometry analysis confirmed that this chemokine restoration drove a re‐infiltration of Ly6G‐positive neutrophils into the skin lesions of the *Mettl1*‐deficient mice (Figure 6F,G).

Western blot analysis demonstrated that BDKRB1 activation effectively restored the previously diminished p38 MAPK phosphorylation in *Mettl1*‐deficient mice, without significantly altering the activation status of ERK or JNK (Figure 6I and Figure S7B). The observation that pharmacological activation yielded a significant but incomplete rescue suggests that the absolute abundance of the BDKRB1 protein is a primary limiting factor in this inflammatory cascade.

### 2.7 In Vivo Restoration of *Bdkrb1* Effectively Rescues Psoriatic Inflammation in *Mettl1*‐Deficient Mice

**FIGURE 7:**
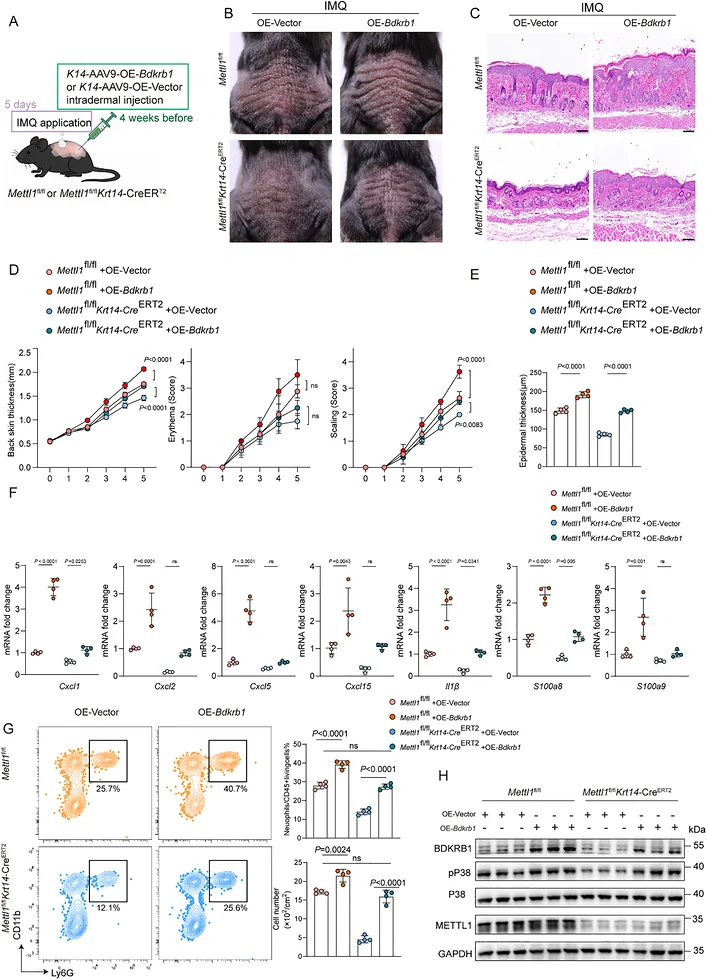
Ectopic in vivo overexpression of *Bdkrb1* fully reverses the protective phenotype of *Mettl1* ablation. (A) Schematic illustration of the in vivo genetic rescue experiment. *Mettl1* ^fl/fl^ and *Mettl1* ^fl/fl^ *Krt14*‐Cre^ERT2^ mice were intradermally injected with K14‐AAV9‐Vector or K14‐AAV9‐OE‐*Bdkrb1* 4 weeks prior to a 5‐day IMQ application. (B) Representative macroscopic photographs of mouse back skin. (C) Representative hematoxylin and eosin (H&E) staining of skin sections. Scale bars = 100 µm. (D) Clinical scoring of back skin thickness, erythema, and scaling for mice treated with IMQ for 5 consecutive days. (*n* = 4 per group). (E) Epidermal thickness, measured from H&E‐stained images. (*n* = 4 per group). (F) mRNA fold change of key neutrophil chemokines (*Cxcl1*, *Cxcl2*, *Cxcl5*, *Cxcl15*) and proinflammatory markers (*Il1b*, *S100a8*, *S100a9*) in the mouse epidermis across the indicated groups, determined by qPCR. (*n* = 4 per group). (G) Representative flow cytometry plots and statistical quantification of the percentage and absolute number of infiltrating neutrophils in mouse skin tissues. (*n* = 4 per group). (H) Western blot analysis of BDKRB1, phosphorylated and total p38, alongside METTL1, in the mouse epidermis across the indicated groups. (*n* = 4 per group). GAPDH was used as the loading control. Data are representative of three independent experiments and are shown as mean ± SD. Statistical significance was determined by one‐way analysis of variance with Tukey's *post hoc* test (D, E, F, G). ns, not significant.

As established above, pharmacological activation yielded a partial rescue because *Mettl1* ablation intrinsically restricted the available pool of BDKRB1 receptors. To overcome this limitation and further validate the METTL1–BDKRB1–p38 axis, we genetically restored Bdkrb1 expression in vivo. Specifically, we utilized a keratinocyte‐specific adeno‐associated virus (K14‐AAV9‐OE‐*Bdkrb1*) to achieve ectopic *Bdkrb1* overexpression in both *Mettl1* ^fl/fl^ and *Mettl1* ^fl/fl^ *Krt14*‐Cre^ERT2^ mice prior to IMQ treatment (Figure 7A).

In *Mettl1* ^fl/fl^ mice, forced *Bdkrb1* overexpression further exacerbated the severity of the IMQ‐induced disease. In *Mettl1*‐deficient mice, replenishing the *Bdkrb1* transcript substantially reversed the protective effects of *Mettl1* ablation. The macroscopic disease severity—reflecting back skin thickness, erythema, and scaling—was restored to levels comparable to the *Mettl1* ^fl/fl^ control group (Figure 7B,D). This phenotypic reversal was confirmed histologically, showing epidermal hyperplasia approaching that of the controls (Figure 7C,E).

At the molecular level, ectopic *Bdkrb1* overexpression re‐established the inflammatory cascade. qPCR analysis revealed that the diminished expression of key neutrophil‐attracting chemokines (*Cxcl1, Cxcl2, Cxcl5, Cxcl15*) and critical proinflammatory markers (*Il1b, S100a8, S100a9*) was effectively restored (Figure 7F), and ELISA confirmed the recovery of chemokine protein secretion (Figure S7C). Flow cytometry confirmed that the infiltration of Ly6G‐positive neutrophils was re‐established in the epidermis of the *Mettl1*‐deficient mice (Figure 7G).

Finally, Western blot analysis confirmed that the AAV‐mediated delivery successfully replenished the BDKRB1 protein pool. This protein restoration correlated closely with the reactivation of p38 MAPK phosphorylation in the *Mettl1*‐ablated epidermis (Figure 7H). Collectively, these in vivo genetic rescue data provide compelling evidence that the depletion of BDKRB1 serves as a primary mechanism by which METTL1 deficiency alleviates psoriatic inflammation.

### 2.8 Dual Pharmacological Blockade of the METTL1–BDKRB1 Axis Synergistically Alleviates Psoriatic Inflammation

**FIGURE 8:**
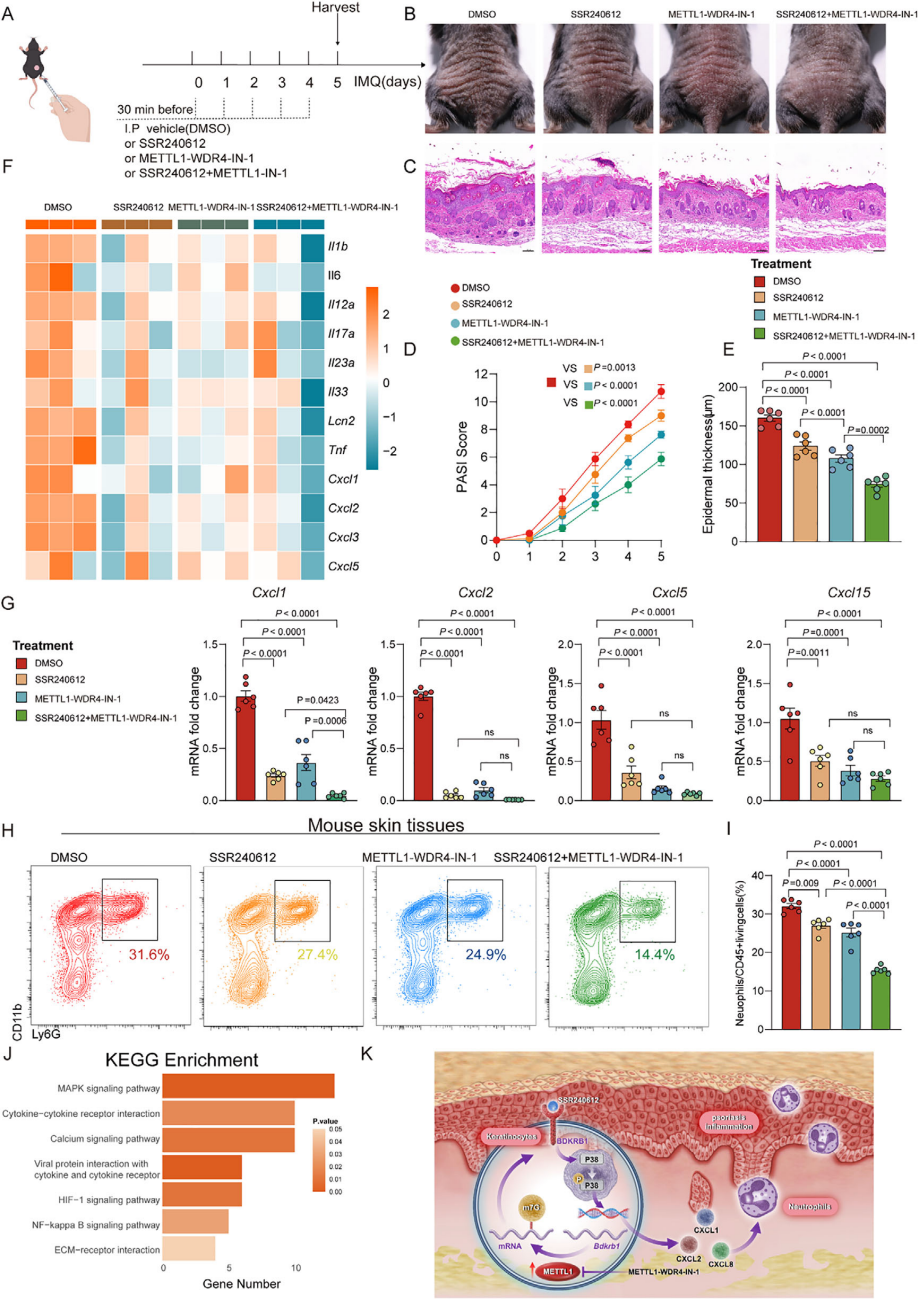
Dual pharmacological blockade of the METTL1–BDKRB1 axis synergistically attenuates psoriasiform inflammation. (A) Schematic representation of the in vivo therapeutic intervention. Mice were intraperitoneally injected with vehicle (DMSO), SSR240612, METTL1‐WDR4‐IN‐1, or their combination, 30 min prior to daily IMQ applications. (B) Representative macroscopic photographs of mouse back skin. (C) Representative hematoxylin and eosin (H&E) staining of skin sections. Scale bars = 100 µm. (D) Cumulative Psoriasis Area and Severity Index (PASI) scores evaluated over the 5‐day IMQ treatment period (*n* = 6 per group). (E) Epidermal thickness, measured from H&E‐stained images (*n* = 6 per group). (F) Heatmap of RNA‐seq data showing the normalized expression (Z‐score) of key proinflammatory cytokines and chemokines across the four treatment groups. (*n* = 3 per group). (G) mRNA fold change of *Cxcl1*, *Cxcl2*, *Cxcl5*, and *Cxcl15* in the mouse epidermis, determined by qPCR. (*n* = 6 per group). (H, I) Representative flow cytometry plots (H) and statistical quantification (I) of infiltrating neutrophils in mouse skin tissues (*n* = 6 per group). (J) KEGG pathway enrichment analysis based on differentially expressed genes across the treatment groups, highlighting the inhibition of the MAPK signaling pathway. (K) Schematic working model of the study. In keratinocytes, the METTL1–WDR4 complex installs m7G modifications on the 5′ UTR of *Bdkrb1* mRNA, thereby enhancing its transcript stability. The upregulated BDKRB1 receptor triggers the phosphorylation of p38 MAPK, which subsequently promotes the robust secretion of CXCL chemokines (CXCL1/2/8). These chemokines recruit neutrophils to the inflammatory site, exacerbating psoriatic inflammation. Pharmacological inhibition of METTL1 and/or BDKRB1 effectively blocks this signaling cascade, mitigating the disease phenotype. Data are representative of three independent experiments and are shown as mean ± SD. Statistical significance was determined by one‐way analysis of variance with Tukey's *post hoc* test (D, E, G, I). ns, not significant.

To investigate the translational therapeutic potential of targeting the newly identified METTL1–BDKRB1 signaling axis, we assessed the in vivo efficacy of the specific METTL1 inhibitor (METTL1‐WDR4‐IN‐1) and the BDKRB1 antagonist (SSR240612) in the IMQ‐induced psoriasis model (Figure 8A).

Relative to vehicle (DMSO)‐treated controls, mice receiving either the METTL1 inhibitor or the BDKRB1 antagonist as a monotherapy exhibited substantially attenuated psoriatic inflammation, evidenced by reduced macroscopic disease severity (PASI scores) and decreased epidermal hyperplasia (Figure 8B–E). Notably, the combined administration of both inhibitors yielded a synergistic therapeutic effect, conferring superior protection against skin inflammation compared with either monotherapy alone.

At the transcriptome level, RNA‐seq analysis of the epidermal tissues demonstrated that all treatment regimens effectively downregulated the expression of cutaneous pathogenic factors (Figure 8F). Specifically, the broad reductions in neutrophil‐attracting *Cxcl* chemokines were further validated by qPCR, which revealed that the combination therapy led to the most profound suppression of *Cxcl1*, *Cxcl2*, *Cxcl5*, and *Cxcl15* (Figure 8G). ELISA of serum corroborated these findings at the protein level (Figure S7D). Correspondingly, flow cytometry analysis corroborated these molecular findings, confirming that cutaneous neutrophil infiltration was most significantly restricted in the combination therapy group (Figure 8H,I).

To ensure that these phenotypic improvements were driven by the intended mechanistic blockade, we examined the downstream signaling cascades. KEGG enrichment analysis of the RNA‐seq data indicated that the MAPK signaling pathway was among the most significantly inhibited pathways across the treatment strategies (Figure 8J). This was functionally confirmed by Western blot analysis of epidermal lysates, which demonstrated that the dual inhibition of METTL1 and BDKRB1 resulted in the most effective suppression of p38 MAPK phosphorylation (Figure S7E,F).

Collectively, these data support a comprehensive working model (Figure 8K) in which METTL1‐mediated m7G modification stabilizes *Bdkrb1* mRNA in keratinocytes, leading to BDKRB1 receptor upregulation and subsequent p38 MAPK activation. This signaling cascade ultimately drives robust CXCL chemokine secretion and massive neutrophil chemotaxis. Crucially, our findings demonstrate that dual pharmacological targeting of this METTL1–BDKRB1 axis represents a highly effective, mechanistically grounded therapeutic strategy for the intervention of psoriatic inflammation.

## 3 Conclusions

This study is the first to systematically define the functional role of epidermal METTL1‐driven m7G modification in psoriasis, extending the known biology of this modification beyond its established roles in tRNA biogenesis, translational regulation, and tumorigenesis into the realm of chronic inflammatory skin disease. We found that METTL1 and m7G levels are elevated in psoriatic lesions, and that inflammatory stimuli directly induce METTL1 expression in keratinocytes. We identified METTL1 as a novel upstream regulator essential for the robust induction of CXCL1, CXCL2, and CXCL8 via stabilization of *Bdkrb1* mRNA through 5' UTR m7G modification, a mechanistically specific post‐transcriptional mechanism rather than a global transcriptome‐wide effect. Epidermal *Mettl1* ablation markedly reduced neutrophil infiltration and attenuated psoriasis severity, whereas its overexpression exacerbated these features, and in vivo genetic restoration of Bdkrb1 fully rescued the psoriatic phenotype in Mettl1‐deficient mice. Critically, dual pharmacological blockade of METTL1 and BDKRB1 produced synergistic suppression of psoriasiform inflammation, most profoundly inhibiting CXCL chemokine expression and cutaneous neutrophil infiltration, providing proof‐of‐concept for this axis as a tractable therapeutic target. These findings collectively establish the METTL1–m7G–BDKRB1–p38 cascade as a critical epitranscriptomic checkpoint governing keratinocyte‐driven neutrophil chemotaxis in psoriasis.

Psoriasis is a complex inflammatory dermatosis where the interplay between epidermal keratinocytes and immune cells choreographs the disease phenotype. While the epitranscriptomic landscape has recently emerged as a pivotal regulator of immune homeostasis [38], previous studies have established that m6A exerts a dichotomous, cell‐type‐dependent function. It acts as a restraint on inflammation in keratinocytes [23], while it functions as a pro‐inflammatory driver in macrophages [24]. By contrast, our study reveals that m7G modification, catalyzed by the METTL1 and WDR4 complex, operates through a fundamentally distinct mechanism in psoriatic keratinocytes: rather than exerting context‐dependent opposing effects, m7G consistently stabilizes pro‐inflammatory transcripts, exemplified here by Bdkrb1 mRNA, to amplify keratinocyte‐driven neutrophil recruitment. This functional divergence between m7G and m6A is not unique to psoriasis. In acute kidney injury (AKI), a paradigmatic systemic inflammatory condition, m6A modifications similarly exhibit opposing effects depending on the writer involved: METTL3‐mediated m6A stabilizes TAB3 mRNA via IGF2BP2‐dependent mechanisms to promote renal inflammation and tubular injury [39], whereas METTL14‐mediated m6A modification of YAP1 mRNA suppresses YAP1 protein expression to aggravate ischemia‐reperfusion injury through a mechanistically distinct pathway [40]. In contrast, m7G modification in AKI converges more consistently on transcript stabilization as a pro‐inflammatory output, with METTL1 stabilizing TEAD2 mRNA in tubular epithelial cells to impair mitochondrial function [41] and Sarm1 mRNA in macrophages to drive NAD+ imbalance and systemic inflammation [31]. Collectively, these cross‐disease observations suggest that while m6A acts as a context‐dependent rheostat whose directionality is determined by the specific reader proteins recruited, m7G tends to function as a more directionally consistent amplifier of pro‐inflammatory mRNA stability. Notably, while this cross‐disease comparison draws on AKI—a systemic inflammatory condition in which both m7G and m6A have been mechanistically dissected—direct functional comparisons between m7G and m6A in other inflammatory skin diseases, such as atopic dermatitis and rosacea, are currently lacking. Whether the directional consistency of m7G observed in psoriasis and AKI extends to other inflammatory dermatoses remains an important open question for future investigation.

However, our understanding of other RNA modifications in inflammatory dermatosis, such as N7‐methylguanosine (m7G), is still in its infancy. Unlike its well‐documented roles in enhancing tRNA stability and translational efficiency during tumorigenesis and embryonic development [42, 43, 44], its potential function in modulating inflammatory responses, especially within the complex milieu of chronic inflammatory diseases like psoriasis, has remained largely unexplored territory. Our study discovered that METTL1 and m7G levels are elevated in psoriatic lesions and that inflammatory stimuli directly induce METTL1 expression in keratinocytes, positioning m7G modification as a previously unrecognized, pro‐inflammatory epitranscriptomic checkpoint in the skin. While m7G is classically known as a 5' cap modification, our study specifically highlights the regulatory role of 5' UTR m7G modification on *Bdkrb1* mRNA stability, consistent with the function of the METTL1‐WDR4 complex. It is noteworthy that while our functional interventions specifically targeted METTL1 as the indispensable catalytic core, the concurrent upregulation of its obligate structural adaptor, WDR4, in psoriatic lesions further reflects the robust hyperactivation of the entire m7G methyltransferase holoenzyme during disease progression.

Neutrophilic inflammation is a characteristic feature of psoriasis pathology [9]. Elevated numbers of neutrophils are found in psoriatic lesions, forming Munro's microabscesses within the stratum corneum and correlating with disease severity [10]. This massive influx of neutrophils is orchestrated by keratinocyte‐derived chemokines, particularly the ELR+ CXC chemokines CXCL1, CXCL2, and CXCL8, which signal through CXCR1/CXCR2 [4, 8, 12, 45]. The importance of this chemokine axis is underscored by its role in other inflammatory diseases like IBD, multiple sclerosis, and rheumatoid arthritis [15, 46, 47]. While the role of these chemokines as downstream effectors in psoriasis is well‐established, the upstream regulatory mechanisms within keratinocytes remain less clear [48]. Our research identifies METTL1 as a novel upstream regulator essential for the robust induction of those chemokines. We demonstrated that depleting METTL1 in keratinocytes markedly reduced neutrophil infiltration and attenuated disease severity, whereas its overexpression exacerbated these features. We acknowledge that the same anti‐Ly6G clone was utilized for both depletion and detection, which may present an epitope masking issue. However, the significant reduction in inflammatory cell infiltration observed in H&E‐stained sections corroborates the effective depletion of neutrophils. This positions METTL1‐mediated m7G modification as a critical control point governing keratinocyte‐driven neutrophil chemotaxis in psoriasis. It should be noted, however, that neutrophilic infiltration is most prominent in acute and pustular psoriasis, whereas the initiation and chronic recurrence of psoriasis vulgaris are more tightly governed by DC–T cell interactions and the IL‐23/Th17 axis [49]. This distinction represents an important limitation of the present study, as our model predominantly captures the METTL1–BDKRB1–CXCL axis and its downstream neutrophil recruitment. Consistently, supplementary flow cytometric analysis revealed that epidermal *Mettl1* ablation did not significantly alter the CD4+/CD8+ T cell ratio in the skin, suggesting that METTL1 deficiency in keratinocytes selectively impairs neutrophil recruitment without broadly disrupting adaptive immune infiltration. Whether METTL1‐driven keratinocyte signaling indirectly shapes the DC–T cell axis through sustained cytokine production, and whether the METTL1–BDKRB1 axis plays a more prominent pathogenic role in pustular psoriasis, where neutrophilic inflammation is the defining feature, warrant future investigation.

BDKRB1, part of the kinin‐kallikrein system, is known for its proinflammatory roles in conditions such as allergic reactions, hereditary angioedema, and rheumatoid arthritis [50, 51, 52]. Kinin signaling has been shown to play a crucial role in neutrophil recruitment, as neutrophil chemotaxis is inhibited in mice lacking the bradykinin B2 receptor with acute inflammation [53]. Although previous studies have hinted at the kinin system's involvement in psoriasis by showing that broad bradykinin receptor blockade ameliorates IMQ‐induced inflammation [54, 55], the specific contributions of BDKRB1 and its regulation remained unknown. Our work provides direct evidence linking METTL1 to BDKRB1 regulation in psoriasis, as we showed that METTL1 enhances *Bdkrb1* mRNA stability via the m7G modification. Crucially, forced in vivo overexpression of *Bdkrb1* in *Mettl1*‐deficient mice completely reversed the protective phenotype and restored massive neutrophil infiltration, confirming that BDKRB1 mediates the inflammatory response as an indispensable METTL1 downstream effector. We further established that this axis converges on the p38 MAPK pathway, a known transducer of inflammatory signals in keratinocytes that regulates chemokine expression [56, 57]. Beyond acute inflammation, the METTL1–BDKRB1 axis may also contribute to psoriasis recurrence. Intriguingly, METTL1 protein expression remained significantly elevated above baseline levels 30 days after the initial IMQ challenge, suggesting that METTL1‐driven stabilization of *Bdkrb1* may persist subclinically during remission. BDKRB1 expression is further sustained by pro‐inflammatory mediators such as IL‐1β and TNF‐α that are known to persist at low levels after apparent disease resolution, potentially maintaining a residual inflammatory state that lowers the threshold for re‐exacerbation upon subsequent triggers. Given the established links between kinin system dysregulation and psoriatic comorbidities, including psoriatic arthritis [58] and cardiovascular disease [59, 60], whether this sustained METTL1–BDKRB1 activity contributes to systemic inflammatory burden warrants future investigation.

The elucidation of the METTL1–BDKRB1–p38 axis offers a compelling rationale for the development of novel therapeutic strategies for psoriasis. Current biologics, although effective, target downstream cytokines and have limitations such as high costs and immunosuppression risks [61, 62]. The targeting of upstream regulators, particularly epitranscriptomic modifiers, is an attractive alternative. The clinical development of METTL3 inhibitors validates the tractability of RNA methyltransferase targeting [63]. Our findings suggest that the inhibition of METTL1 in keratinocytes could be a more targeted and safer approach. The synergistic effect observed with combined METTL1 and BDKRB1 inhibition is particularly promising, as it suggests that this dual targeting could effectively disrupt the keratinocyte‐driven inflammatory loop, potentially leading to the development of more effective small‐molecule therapies for psoriasis. While promising, the in vivo efficacy and potential toxicity of long‐term dual inhibition of this pathway remain to be fully investigated.

Although our findings provide novel insights, further investigation is warranted. The validation of our results with psoriasis models not based on IMQ induction, such as those involving IL‐23 injection or spontaneous induction, will be important. Furthermore, the exploration of the role of METTL1 in other skin cells, such as fibroblasts and endothelial cells, could reveal broader functions. Finally, the development of clinical‐grade METTL1 inhibitors suitable for dermatological use is a necessary next step for translation.

In conclusion, we identified a previously unrecognized epitranscriptomic pathway that is critical for psoriatic inflammation. We demonstrated that METTL1 is upregulated in psoriatic keratinocytes and promotes inflammation by stabilizing *Bdkrb1* mRNA via m7G methylation. This process leads to enhanced BDKRB1–p38 signaling and neutrophil chemoattractant production, and consequently to amplified neutrophil infiltration. These findings extend the known biology of m7G modification beyond its established roles in tRNA biogenesis, translational regulation, and tumorigenesis into chronic inflammatory skin disease, enriching the theoretical framework of epigenetic and epitranscriptomic regulation in psoriasis pathogenesis. The results suggest that METTL1 and BDKRB1 are tractable therapeutic targets for psoriasis, with BDKRB1's inducible and disease‐restricted expression profile conferring a favorable therapeutic window. They open a new avenue for epitranscriptomic‐based intervention in inflammatory skin disease and potentially in other neutrophil‐driven inflammatory conditions.

## 4 Experimental Section

### 4.1 Human Subjects

Samples were collected from human subjects for this study at the First Affiliated Hospital of Anhui Medical University, Hefei, China. Peripheral blood was acquired from healthy volunteers and patients with psoriasis vulgaris. Two senior dermatologists independently confirmed all psoriasis vulgaris diagnoses. Paraffin‐embedded specimens of healthy skin and paired psoriatic lesional and non‐lesional skin were provided by the hospital's Dermatology Pathology Laboratory. Written informed consent was obtained from each participant before inclusion.

### 4.2 Mice

GemPharmatech Corporation (Nanjing, China) supplied C57BL/6J, *Mettl1^flox/flox^*, and keratin 14 (Krt14)–Cre‐estrogen receptor (ER) T2 transgenic mice. All animals were maintained at the Laboratory Animal Center of Anhui Medical University in a specific pathogen–free facility. The environment was controlled with a 12/12‐h light/dark cycle, a temperature of 22°C–25°C, and humidity of 30–70%. Eight‐ to 10‐week‐old female mice on the C57BL/6 background were utilized for specific protocols.

### 4.3 Treatment of Mice

To evaluate the in vivo contribution of the epidermal m7G modification, we created an inducible keratinocyte‐specific *Mettl1*‐deficient mouse model by breeding *Mettl1^flox/flox^* mice with Krt14‐Cre^ERT2^ mice (Figure 2A and Figure S3A). *Mettl1* deletion was induced in 5‐week‐old mice via five consecutive daily intraperitoneal injections of tamoxifen (#T5648; Sigma–Aldrich) formulated in corn oil (75 µg/g body weight). Nine days after the final injection, KO efficiency was verified.

An imiquimod (IMQ)‐induced psoriasiform dermatitis model was established using *Mettl1^fl/fl^Krt14‐Cre^ERT2^* mice and their *Mettl1^fl/fl^* littermates. Psoriasis‐like skin inflammation was initiated by the daily topical application of 62.5 mg 5% IMQ cream (Med‐shine Pharma) to a 2.5 × 2.5 cm shaved dorsal skin patch on each mouse for five consecutive days. The clinical severity of skin inflammation was evaluated daily using a modified Psoriasis Area and Severity Index (PASI) scoring system. Erythema, scaling, and skin thickening were scored independently on a scale from 0 to 4: 0, none; 1, slight; 2, moderate; 3, marked; and 4, very marked. The mice were subjected to isoflurane‐induced anesthesia and euthanized by cervical dislocation.

For neutrophil clearance, 7‐week‐old *K14‐*AAV9‐OE*‐Mettl1* mice received rat anti‐mouse Ly6G antibody (#BP0075‐1; BioXCell) or control rat immunoglobulin G (IgG; #BP0089; BioXCell). The initial dose (400 µg/20 g body weight) was given 1 day prior to IMQ treatment, and maintenance doses (100 µg/20 g body weight) were administered every other day starting 1 day after each IMQ application. This depletion protocol was adopted from a previously established method by Cui et al. [23], which demonstrated effective neutrophil clearance in a similar experimental setting.

To achieve keratinocyte‐specific overexpression of *Mettl1* or *Bdkrb1* in vivo, adeno‐associated virus serotype 9 (AAV9) vectors carrying the respective genes under the control of the K14 promoter (K14‐AAV9‐OE‐*Mettl1* and K14‐AAV9‐OE‐*Bdkrb1*) alongside empty vectors (K14‐AAV9‐Vector) were constructed. Mice received multi‐point intradermal injections of the AAV solutions into the shaved dorsal skin 4 weeks prior to the IMQ application to ensure sufficient epidermal expression.

For in vivo pharmacological inhibition, mice were intraperitoneally injected with 0.2 mg of the METTL1 inhibitor METTL1‐WDR4‐IN‐1 (MedChemExpress, #HY‐162080), 0.2 mg of the BDKRB1 antagonist SSR240612 (MedChemExpress, #HY‐15039), a combination of both, or 200 µl of 10% DMSO (vehicle control) 30 min prior to daily IMQ treatment. The dosage of METTL1‐WDR4‐IN‐1 was selected based on a recent study by Wang et al. [28], which validated its inhibitory effect on m7G modification. The dose of SSR240612 was determined according to Soley et al. [55], who reported that this concentration effectively improves psoriasis‐like symptoms.

### 4.4 Dual‐Luciferase Reporter Assay

To assess the regulatory role of 5' UTR m7G methylation on *BDKRB1* stability, the wild‐type (WT) and mutant (MUT) *BDKRB1* 5' UTR sequences were synthesized and cloned into pmirGLO dual‐luciferase reporter vectors. HaCaT cells were co‐transfected with the reporter plasmids and shNC or sh*METTL1* using Lipofectamine 3000. Following M5 cytokine stimulation, firefly and Renilla luciferase activities were measured using a Dual‐Luciferase Reporter Assay System (Promega), and relative activity was calculated.

### 4.5 Cell Line and Culture

The HaCaT immortalized human keratinocyte line was obtained from the National Collection of Authenticated Cell Cultures (# SCSP‐5091). All cells were routinely tested and found negative for mycoplasma contamination. Cells between passages 5 and 10 were maintained in Dulbecco's modified Eagle medium supplemented with 10% fetal bovine serum and 1% antibiotics. Cells were stimulated with an M5 cytokine cocktail consisting of recombinant human IL‐1α (#20001A; PeproTech), IL‐17A (#200‐17; PeproTech), IL‐22 (#200‐22; PeproTech), oncostatin M (#300‐10; PeproTech), and TNF‐α (#300‐01A; PeproTech; all 10 ng/ml). Lipofectamine 3000 (#L3000075; Invitrogen) was used for small interfering (si)RNA transfection. The sequences are provided in Table S1.

### 4.6 Histological and Immunofluorescence Analyses

Skin specimens were stained with hematoxylin and eosin for morphological assessment. Epidermal thickness was quantified at randomly chosen sites using ImagePro Plus 6.0 software.

After deparaffinization, sections underwent antigen retrieval by boiling (15 min) in 10 mm sodium citrate buffer (pH 6.0). Activity from endogenous peroxidases was blocked with 3% hydrogen peroxide. Non‐specific binding was inhibited using a blocking solution (#P0260; Beyotime). Slides were incubated overnight at 4°C with primary anti‐mouse/human METTL1 antibody (#ab271063; 1:200;Abcam), anti‐mouse/human METTL1 antibody (#ab271063; 1:200;Abcam), anti‐mouse/human CXCL1/GRO antibody (#ab322200; 1:200;Abcam), anti‐mouse Ki67 antibody (#ab15580; 1:200;Abcam), anti‐mouse Loricrin antibody (#ab185679; 1:200;Abcam), anti‐mouse Cytokeratin 10 antibody (#ab76318; 1:200;Abcam) and anti‐mouse Filaggrin antibody (#905804; 1:200; BioLegend). An HRP‐conjugated goat anti‐rabbit IgG (ZSGB‐BIO, #ZB‐2301, 1:200) served as the secondary antibody. Signals were visualized using 3,3´‐diaminobenzidine solution (#ZLI‐9017; ZSGB‐BIO).

For immunofluorescence (IF) analysis, sections were blocked as for IHC analysis, but without peroxidase quenching, and then incubated with the following primary antibodies: anti‐METTL1 (#ab271063; Abcam), anti‐Ly6G (#127601; Biolegend), anti‐KRT14 (#74956; CST), and anti‐human BDKRB1 (#YT0514; Immunoway). Appropriate secondary antibodies (#ab150115; Abcam) were used, 1:300 for all antibodies.

### 4.7 RNA Extraction and Real‐Time Quantitative PCR

Total RNA was isolated from epidermis, cultured cells, and whole blood samples with Trizol (#9109; Takara) following the vendor's protocol. The Evo M‐MLV RT kit (#AG11705; Accurate Biology) was used to reverse transcribe RNA into complementary DNA. SYBR Green premix (#AG11762; Accurate Biology) was used for quantitative polymerase chain reaction (qPCR) with a QuantStudio Dx real‐time PCR system (Thermo Fisher). Gene expression was calculated relative to the *GAPDH* housekeeping gene using the 2^−ΔΔCt^ method. All primers, listed in Table S2, were synthesized by Sangon Biotech.

### 4.8 Western Blot Analysis

Protein was extracted from epidermis and cell samples using radioimmunoprecipitation assay buffer (#P0013C; Beyotime) supplemented with phenylmethylsulfonyl fluoride (#ST506; Beyotime) and a protease/phosphatase inhibitor cocktail (#P1008; Beyotime). Following quantification, 20 µg of protein from each sample was loaded per lane, resolved on 10% SDS‐PAGE gels, and subsequently transferred to nitrocellulose membranes. The membranes were immunoblotted using the following primary antibodies: anti‐GAPDH (#10494‐1‐AP; 1:5000 Proteintech), anti‐METTL1 (#ab271063; 1:1000 Abcam), anti‐WDR4 (#A19765; 1:1000 Abclonal), anti‐mouse BDKRB1 (#ABR‐013; Alomone Labs), anti‐human BDKRB1 (#YT0514; Immunoway), phosphorylated (p‐) extracellular signal–regulated kinase (ERK) 1/2 (#4370T; CST), ERK1/2 (#4695T; CST), p‐p38 (#4511T; CST), p38 (#8690T; CST), p‐JNK(#4058S; CST), and JNK (#4691S; CST; 1:1000 for all antibodies). Signals were detected using electrochemiluminescence solution (#BMU102; Abbkine) and visualized on a FluorChem FC3 imaging system (ProteinSimple).

### 4.9 RNA Sequencing and m7G Methylated RNA Immunoprecipitation Sequencing

To elucidate the molecular mechanisms by which m7G modification impacts epidermal keratinocytes in psoriasis, we conducted methylated RNA immunoprecipitation sequencing (MeRIP‐seq) alongside RNA‐seq of paired lesional and healthy skin from patients with psoriasis and epidermis from IMQ‐treated *Mettl1^fl/fl^ and Mettl1^fl/fl^Krt14‐Cre^ERT2^* mice (Figure 4A). MeRIP‐seq was performed by CloudSeq Inc. (Shanghai, China). In brief, Trizol‐extracted total RNA was first treated with a decapping enzyme to remove the 5' cap m7G, thereby eliminating cap‐associated background and ensuring the specific enrichment of 5' UTR and internal m7G modifications. The RNA was then fragmented into ∼200‐nt segments. Protein A/G beads were conjugated with an m7G antibody. The RNA fragments were then mixed with the antibody–bead complexes and incubated at 4°C for 4 h. After rigorous washing, the captured RNA was eluted. Libraries for the immunoprecipitated and input control fractions were prepared using the GenSeq Low Input Whole RNA Library Prep Kit. An Agilent 2100 bioanalyzer was used to validate the libraries, which were then sequenced on an MGI DNBSEQ‐T7 platform.

### 4.10 Dot Blot Assay

Total RNA was purified using TRIzol (Takara, #9109). RNA dilutions were applied to a nylon membrane (Beyotime, #FFN13). The membrane was then UV‐cross‐linked and blocked with 5% nonfat milk before an overnight incubation with the Anti‐7‐methylguanosine (m7G)‐Cap mAb(1:1000, # RN017M, MBL Life Science). To visualize total RNA loading, the membrane was stained with 0.02% methylene blue in 0.3 m sodium acetate (pH 5.2).

### 4.11 Enzyme‐Linked Immunosorbent Assay

For serum chemokine measurement in mice, venous blood was collected from all subjects into anticoagulant‐treated EP tubes and centrifuged for 15 min at 3000 rpm at 4°C. The supernatant was carefully collected and stored at −80°C until analysis. For cell culture supernatant chemokine measurement, HaCaT cell culture supernatants were collected following the indicated treatments and centrifuged at 1000 rpm for 5 min to remove cell debris. Chemokine concentrations were determined using enzyme‐linked immunosorbent assay kits for mouse CXCL1 (Mairui #MR‐03093M1), mouse CXCL2 (Mairui #MR‐12004M1), mouse CXCL5 (Mairui #MR‐12015M1), mouse CXCL15 (Mairui #MR‐12015M2), human CXCL1 (Mairui #MR‐1457H1), human CXCL2 (Mairui #MR‐6060H1), and human CXCL8 (Mairui #MR‐10214H1) according to the manufacturers' protocols.

### 4.12 RNA Stability Assay

To halt transcription, HaCaT cells were treated with actinomycin (5 µg/ml). Samples were harvested at 0, 3, and 6 h post‐treatment. *Bdkrb1* expression was quantified by real‐time qPCR following total RNA extraction.

### 4.13 Skin Immune Cell Isolation and Flow Cytometry

For cell isolation, mouse skin was minced finely on ice and digested at 37°C (225 rpm, 1–1.5 h) in Roswell Park Memorial Institute 1640 medium (#11875093; Gibco) containing bovine serum albumin (#4240GR100, 20 mg/ml; Biofroxx), type 4 collagenase (#LS004189, 1 mg/ml; Worthington), 4‐(2‐hydroxyethyl)‐1‐piperazineethanesulfonic acid (#15630080, 1%; Gibco), and DNase I (#D8071, 10 mg/ml; Solarbio). The resulting cell suspension was passed through a 70‐µm strainer (#431751; Corning). Immune cells were isolated from the interface of a 30%/70% lymphocyte separation medium gradient (2000 rpm, 30 min), washed, and resuspended in phosphate‐buffered saline with serum albumin (20 mg/ml) for counting.

For flow cytometry, single‐cell suspensions were first incubated with cluster of differentiation (CD)16/CD32 monoclonal antibody (#16‐0161‐82; 1:300;eBioscience) for Fc receptor blocking. Staining was performed using fixable viability stain 510 (#564406, 1:300; BD Biosciences), allophycocyanin (APC)‐cyanine 7 (Cy7) rat anti‐mouse CD45 (#557659, 1:200; BD Biosciences), brilliant violet 421 rat anti‐CD11b (#562605, 1:200; BD Biosciences), phycoerythrin (PE) hamster anti‐mouse CD11c (#557401, 1:200; BD Biosciences), APC rat anti‐mouse Ly6G (#560599, 1:200; BD Biosciences), fluorescein isothiocyanate rat anti‐mouse Ly6C (#553104, 1:200; BD Biosciences), and PE/Cy7 anti‐mouse F4/80 (#123114, 1:200; Biolegend). The proportions of neutrophils, macrophages, monocytes, and dendritic cells (DCs) were determined. For T cell analysis, a separate panel was used: APC‐Cy7 anti‐mouse CD45 (#103116, 1:200; BioLegend), PerCP‐Cy5.5 anti‐mouse CD3 (#560527, 1:200; BD Biosciences), Brilliant Violet 421 anti‐mouse CD4 (#740008, 1:200; BD Biosciences), and PE anti‐mouse CD8a (#553032, 1:200; BD Biosciences). The proportions and absolute numbers of CD4^+^ and CD8^+^ T cells were determined. A CytoFLEX device (Beckman Coulter) was used for data acquisition, and FlowJo (v10.8.1) was used for analysis.

### 4.14 Ethical Considerations

All procedures involving animals followed the Guide for the Care and Use of Laboratory Animals and received clearance from the Laboratory Animal Ethics Committee of Anhui Medical University (no. LLSC20232224). Comprehensive measures were taken to minimize animal suffering. The human study protocol was approved by the Biomedical Ethics Committee of Anhui Medical University (no. 20190195), and all procedures were conducted in compliance with the Declaration of Helsinki and other international ethical guidelines.

### 4.15 Statistical Analysis

All quantitative data were presented as the mean ± standard deviation (SD). No data pre‐processing (e.g., data transformation or outlier removal) was performed prior to analysis unless otherwise specified. Sample sizes (*n*) representing independent biological replicates for each experiment are detailed in the respective figure legends. Statistical analyses were conducted using GraphPad Prism software (version 8.0.2). The assumption of normality was evaluated, and appropriate tests were selected accordingly. For comparisons between two independent groups, an unpaired, two‐tailed Student's *t*‐test was utilized. For comparisons among three or more groups, a one‐way analysis of variance (ANOVA) was performed, followed by Tukey's *post hoc* test for multiple comparisons to adjust the alpha value. A testing level (alpha value) of 0.05 was applied, and a *p*‐value of < 0.05 was considered statistically significant.

## Author Contributions

L.S., Q.Z., and C.Z. contributed to the conceptualization and supervision. C.Z. performed the methodology and formal analysis. C.Z., J.L., and S.Y. carried out the investigation. Y.Y., W.C., Y.H., and Z.Z. were responsible for data curation. J.L. and S.Y. provided animal husbandry resources, and Y.W. and Z.L. provided clinical sample resources. C.Z. wrote the original draft. L.S. and Q.Z. reviewed and edited the manuscript. All authors have read and agreed to the published version of the manuscript.

## Funding

This study was supported by and the National Natural Science Foundation of China (No. 82404152, China) the Science and Technology Program of Hebei (No. 23372505D, China), the Hebei Natural Science Foundation (No. H2023209084, China), the Science and Technology Project of Hebei (No. 252W5202D, China), and the Science Research Project of Hebei Education Department (No. QN2024003, China).

## Conflicts of Interest

The authors declare no conflicts of interest.

## Supporting information




**Supporting file**: advs75970‐sup‐0001‐SuppMat.docx

## Data Availability

The data that support the findings of this study are available from the corresponding author upon reasonable request.
