# Supplementary material for: Epidermal METTL1‐Mediated m7G Modification Drives Psoriatic Inflammation by Stabilizing Bdkrb1 and Orchestrating Neutrophil Recruitment
Source: Adv Sci (Weinh). 2026 Jun 9:e75970. Online ahead of print. doi: 10.1002/advs.75970 (PMC13336495; doi:10.1002/advs.75970)
Supplement: Supplementary file 1 — Supporting file: advs75970‐sup‐0001‐SuppMat.docx [file ADVS-9999-e75970-s001.docx]

Supporting Information

**Epidermal METTL1-Mediated m7G Modification Drives Psoriatic Inflammation by Stabilizing *Bdkrb1* and Orchestrating Neutrophil Recruitment**

*Chang Zhang, Jiayi Lu, Sihao Yan, Yirui Wang , Zhuo Li, Weiwei Chen, Yang Han, Ziyan Zhang, Yafen Yu, Qi Zhen*, Liangdan Sun*.*

**Figure S1**


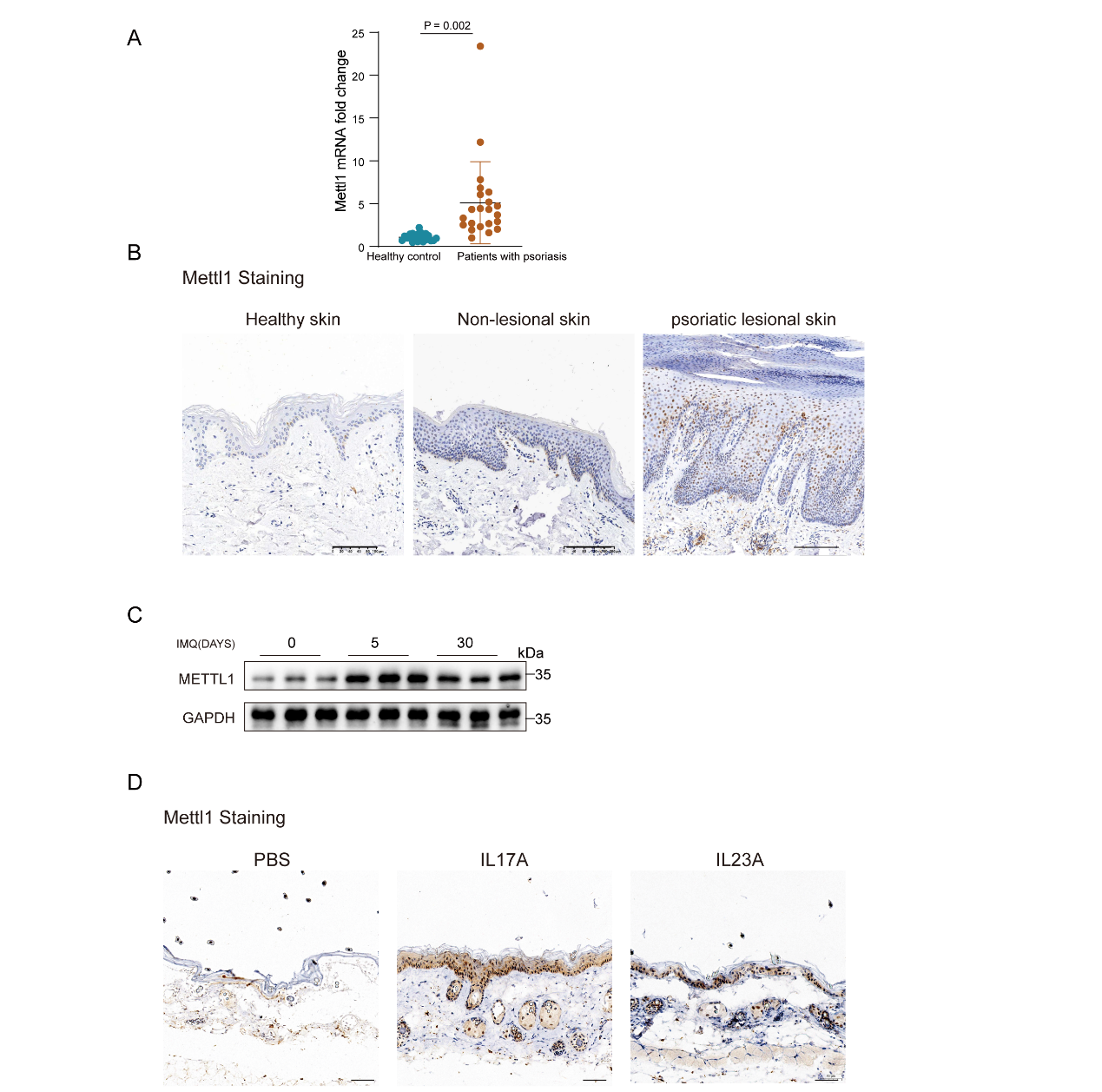


**Figure S1. Expression pattern of METTL1 in human and mouse psoriatic skin.** (A) *METTL1* mRNA expression levels in the peripheral blood of healthy controls and patients with psoriasis. (B) Representative immunohistochemistry (IHC) staining images of METTL1 in human healthy skin, non-lesional skin (PN), and psoriatic lesional skin (PA). (C) Western blot analysis of METTL1 protein expression in the epidermis of mice at Day 0, Day 5 (end of IMQ treatment), and Day 30 post-initial IMQ challenge. GAPDH was used as the loading control. (n = 3 per group). (D) Representative IHC staining images of METTL1 in the skin of mice intradermally injected with PBS, recombinant IL-17A, or IL-23A. Data are presented as means ± standard deviations. Statistical significance was determined by unpaired Student’s t-test (A).

**Figure S2**


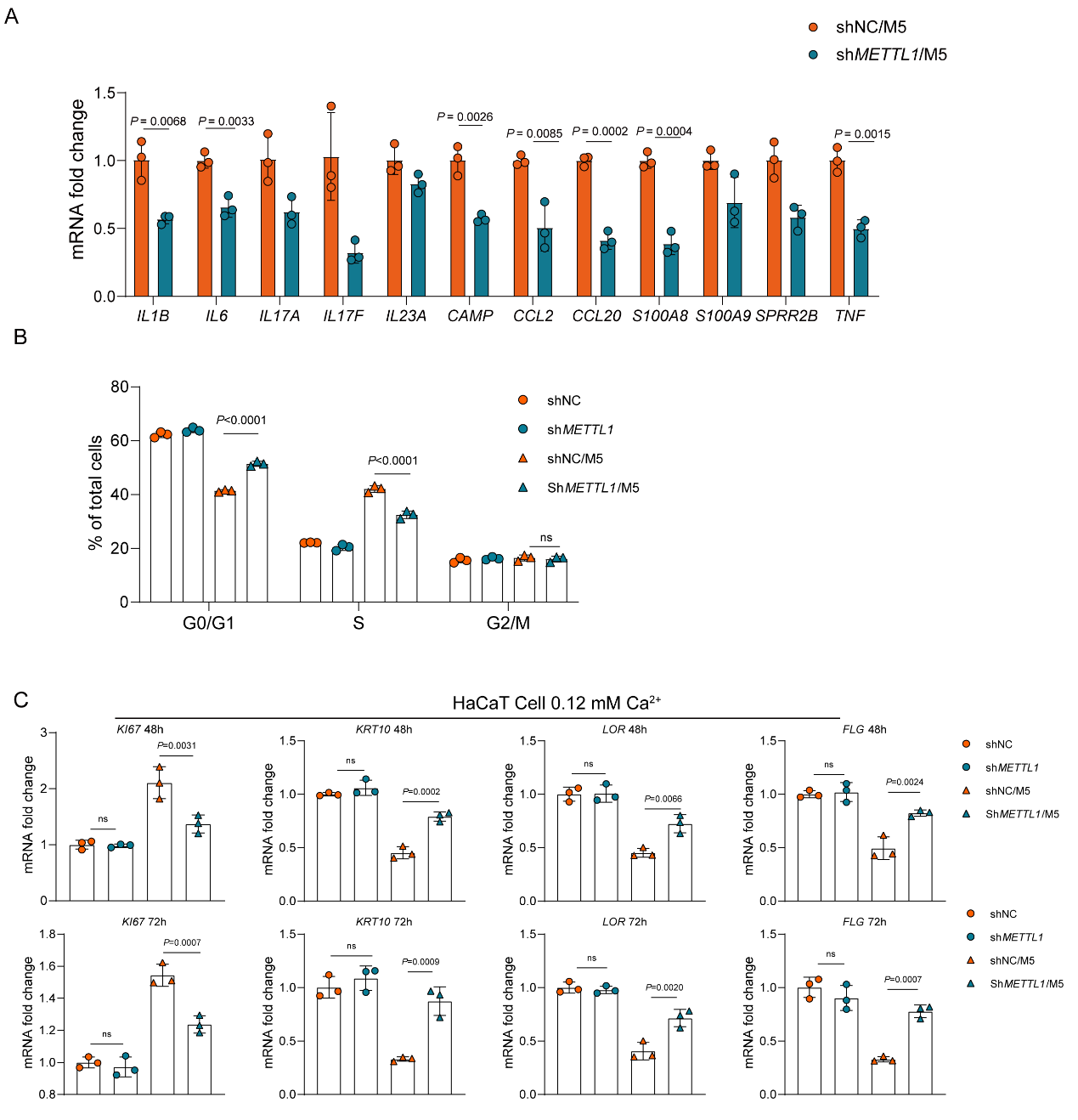


**Figure S2.** *METTL1* knockdown attenuates inflammatory gene expression and alters keratinocyte proliferation and differentiation under inflammatory conditions *in vitro*. (A) qRT-PCR analysis of inflammatory gene expression in shNC and sh*METTL1* HaCaT cells stimulated with M5 cytokine cocktail. (n = 3 per group). (B) Cell cycle distribution analysis by flow cytometry in shNC and sh*METTL1* HaCaT cells with or without M5 stimulation. (n = 3 per group). (C) qRT-PCR analysis of the proliferation marker *KI67* and terminal differentiation markers *KRT10*, *LOR*, and *FLG* in shNC and sh*METTL1* HaCaT cells subjected to 0.12 mM Ca²⁺-induced differentiation with or without M5 stimulation at 48 and 72 hours. (n = 3 per group). Data are representative of three independent experiments and shown as mean ± SD. Statistical significance was determined by unpaired Student’s t-test (A) or one-way analysis of variance with Tukey's *post-hoc* test (B,C). ns, not significant.

**Figure S3**

**
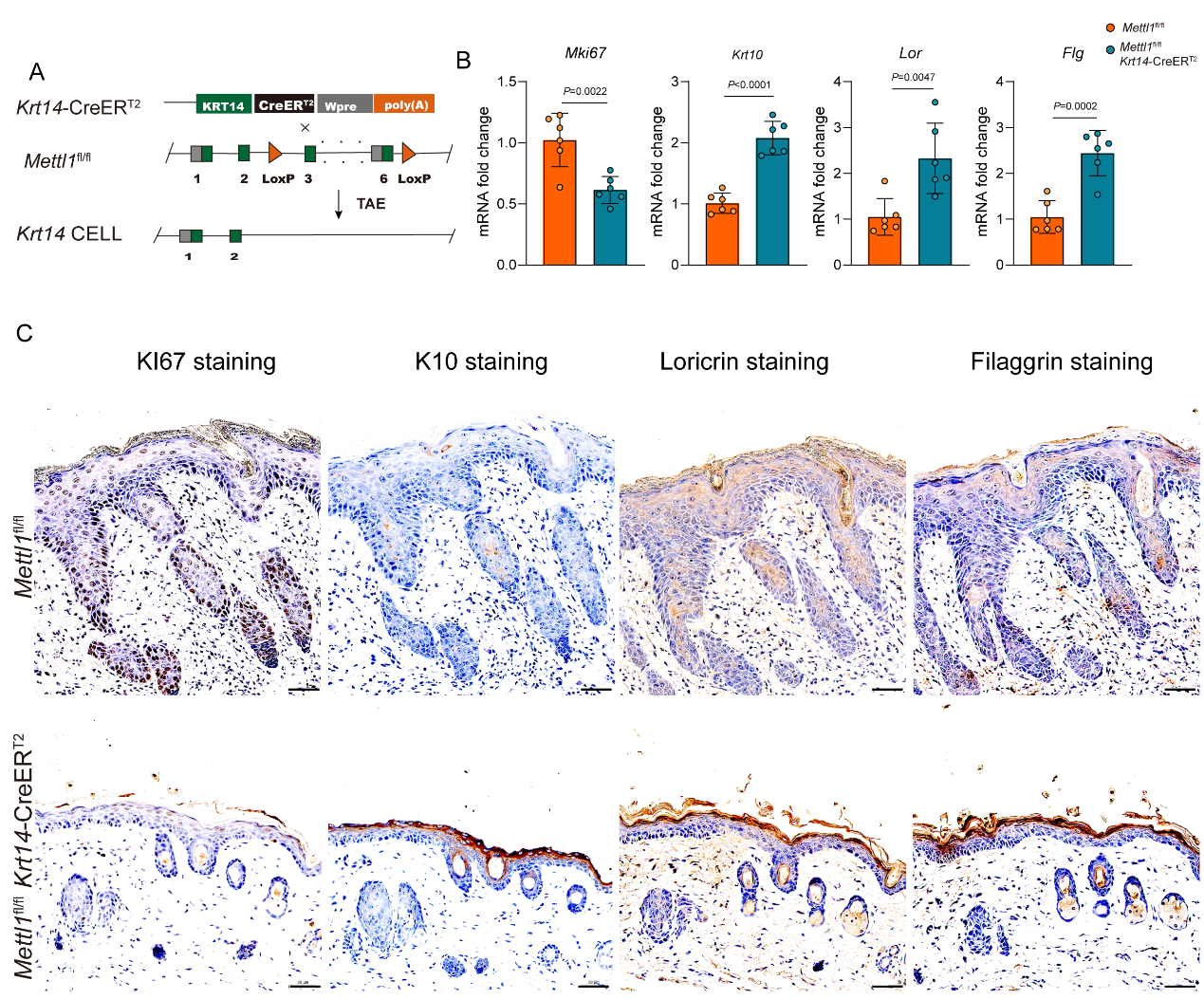
**

**Figure S3.** Epidermal *Mettl1* deficiency reduces keratinocyte proliferation and promotes differentiation *in vivo*. (A) Schematic illustration of the generation of *Mettl1*fl/fl*Krt14*-CreERT2 conditional knockout mice. (B) qRT-PCR analysis of *Mki67*, *Krt10*, *Lor*, and *Flg* mRNA expression in the epidermis of IMQ-treated *Mettl1*fl/fl and *Mettl1*fl/fl*Krt14*-CreERT2 mice. (n = 6 per group). (C) Representative IHC staining images of KI67, K10, Loricrin, and Filaggrin in the skin of IMQ-treated *Mettl1*fl/fl and *Mettl1*fl/fl*Krt14*-CreERT2 mice. Scale bars, 50 μm. Data are representative of three independent experiments and shown as mean ± SD. Statistical significance was determined by unpaired Student’s t-test (B). ns, not significant.

**Figure S4**

**
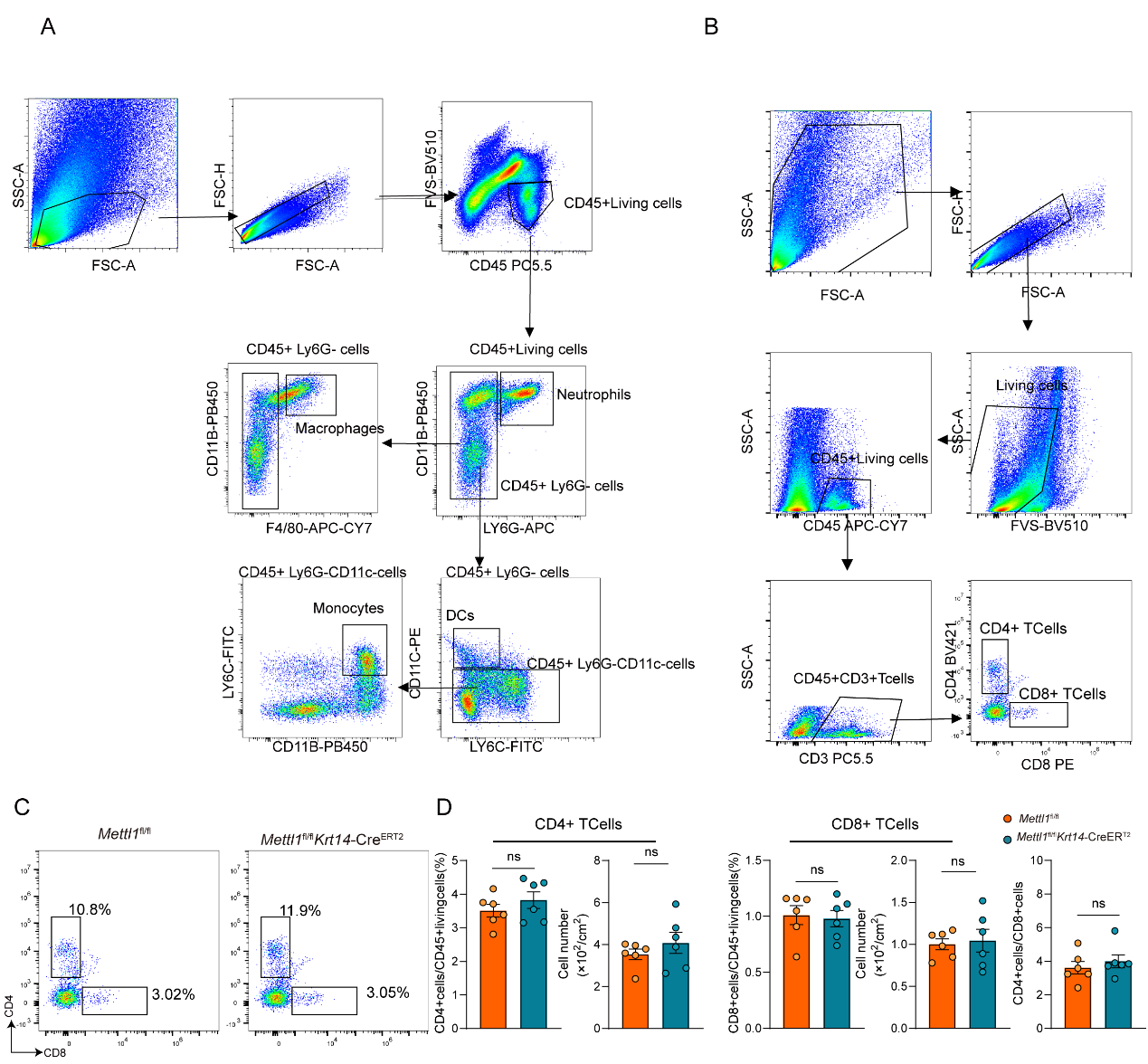
Figure S4.** **Flow cytometry gating strategies and analysis of immune cell infiltration in IMQ-treated mouse skin.** (A) Gating strategy for neutrophils (CD45⁺Ly6G⁺CD11B⁺), macrophages (CD45⁺Ly6G⁻CD11B⁺F4/80⁺), dendritic cells (CD45⁺Ly6G⁻CD11C⁺), and monocytes (CD45⁺Ly6G⁻CD11C⁻CD11B⁺Ly6C⁺). (B) Gating strategy for CD4⁺ and CD8⁺ T cells (CD45⁺CD3⁺CD4⁺ and CD45⁺CD3⁺CD8⁺). (C) Representative flow cytometry plots of CD4⁺ and CD8⁺ T cells in the skin of IMQ-treated *Mettl1*fl/fl and *Mettl1*fl/fl*Krt14*-CreERT2 mice. (D) Quantification of CD4⁺ T cell proportion and absolute number, CD8⁺ T cell proportion and absolute number, and CD4⁺/CD8⁺ ratio (n = 6 per group). Data are representative of three independent experiments and shown as mean ± SD. Statistical significance was determined by unpaired Student’s t-test (D). ns, not significant.

**Figure S5**


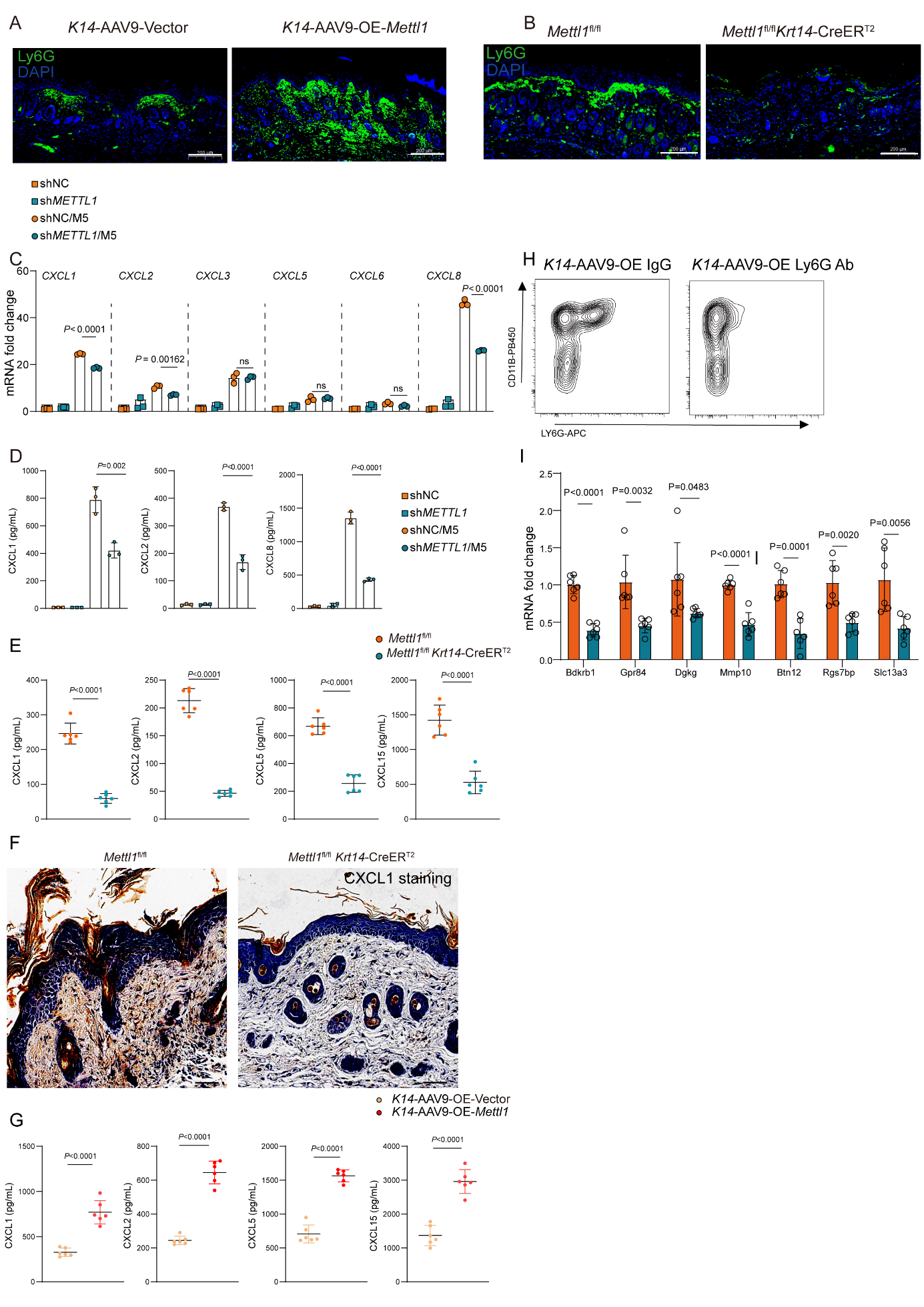


**Figure S5.** **Epidermal METTL1 orchestrates neutrophil recruitment via the CXCL chemokine axis.** (A) Representative immunofluorescence images of Ly6G (green) and DAPI (blue) in the skin of K14-AAV9-Vector and K14-AAV9-OE-*Mettl1* mice after IMQ treatment. Scale bars = 200 μm. (B) Representative immunofluorescence images of Ly6G (green) and DAPI (blue) in the skin of IMQ-treated *Mettl1*fl/fl and *Mettl1*fl/fl*Krt14*-CreERT2 mice. Scale bars = 200 μm. (C) qRT-PCR analysis of *CXCL1*, *CXCL2*, *CXCL3*, *CXCL5*, *CXCL6*, and *CXCL8* mRNA expression in shNC and sh*METTL1* HaCaT cells with or without M5 stimulation (n = 3 per group). (D) ELISA quantification of CXCL1, CXCL2, and CXCL8 protein levels in the culture supernatant of shNC and sh*METTL1* HaCaT cells with or without M5 stimulation (n = 3 per group). (E) ELISA quantification of CXCL1, CXCL2, CXCL5, and CXCL15 protein levels in serum of IMQ-treated *Mettl1*fl/fl and *Mettl1*fl/fl*Krt14*-CreERT2 mice (n = 6 per group). (F) Representative IHC staining images of CXCL1 in the skin of IMQ-treated *Mettl1*fl/fl and *Mettl1*fl/fl*Krt14*-CreERT2 mice. Scale bars = 50 μm. (G) ELISA quantification of CXCL1, CXCL2, CXCL5, and CXCL15 protein levels in serum of IMQ-treated K14-AAV9-Vector and K14-AAV9-OE-*Mettl1* mice (n = 6 per group). (H) Representative flow cytometry plots showing the efficiency of anti-Ly6G antibody-mediated neutrophil depletion in K14-AAV9-OE-*Mettl1* mice compared with isotype IgG-treated controls. (I) qRT-PCR validation of the seven conserved candidate genes (*Bdkrb1*, *Gpr84*, *Dgkg*, *Mmp10*, *Btnl2*, *Rgs7bp*, and *Slc13a3*) in the epidermis of IMQ-treated *Mettl1*fl/fl and *Mettl1*fl/fl*Krt14*-CreERT2 mice (n = 6 per group). Data are representative of three independent experiments and shown as mean ± SD. Statistical significance was determined by unpaired Student's *t*-test (C–G, I). ns, not significant.

**Figure S6**


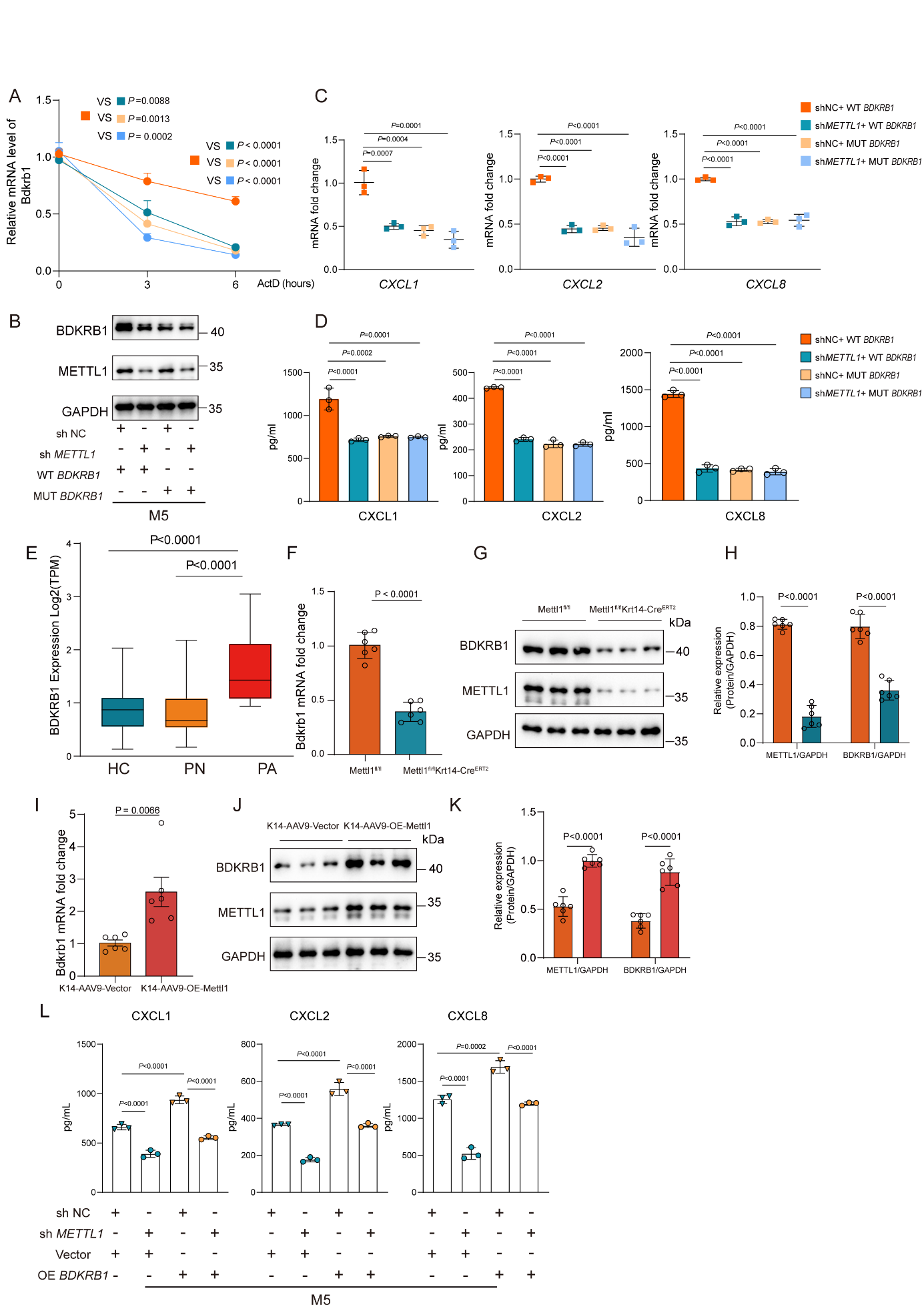


**Figure S6.** Site-directed mutagenesis validates the METTL1–m7G–BDKRB1 regulatory axis and its downstream chemokine output. (A) *BDKRB1* mRNA decay rates in HaCaT cells expressing wild-type (WT) or 5′ UTR-mutated (MUT, GAAGA→CAACA) *BDKRB1* with shNC or sh*METTL1*, following actinomycin D treatment (n = 3 per group). (B) Western blot analysis of BDKRB1 and METTL1 protein expression in the indicated groups under M5 stimulation. GAPDH was used as the loading control. (C) qRT-PCR analysis of *CXCL1*, *CXCL2*, and *CXCL8* mRNA expression in HaCaT cells expressing WT or MUT *BDKRB1* with shNC or sh*METTL1* under M5 stimulation (n = 3 per group). (D) ELISA quantification of CXCL1, CXCL2, and CXCL8 protein levels in the culture supernatant of the corresponding groups under M5 stimulation (n = 3 per group). (E) *BDKRB1* mRNA expression (Log2 TPM) in human healthy control (HC), non-lesional (PN), and psoriatic lesional (PA) skin from the GSE54456 dataset. (F) *Bdkrb1* mRNA expression in the epidermis of IMQ-treated *Mettl1*fl/fl and *Mettl1*fl/fl*Krt14*-CreERT2 mice (n = 6 per group). (G, H) Western blot analysis (G) and quantification (H) of BDKRB1 and METTL1 protein expression in the epidermis of IMQ-treated *Mettl1*fl/fl and *Mettl1*fl/fl*Krt14*-CreERT2 mice (n = 6 per group). GAPDH was used as the loading control. (I) *Bdkrb1* mRNA expression in the epidermis of IMQ-treated K14-AAV9- Vector and K14-AAV9-OE-*Mettl1* mice (n = 6 per group). (J, K) Western blot analysis (J) and quantification (K) of BDKRB1 and METTL1 protein expression in the epidermis of IMQ-treated K14-AAV9-Vector and K14-AAV9-OE-*Mettl1* mice (n = 6 per group). GAPDH was used as the loading control. (L) ELISA quantification of CXCL1, CXCL2, and CXCL8 protein levels in the culture supernatant of HaCaT cells across different rescue conditions (shNC, sh*METTL1*, Vector, OE *BDKRB1*) under M5 stimulation (n = 3 per group). Data are representative of three independent experiments and shown as mean ± SD. Statistical significance was determined by unpaired Student's *t*-test (F, H, I, K) or one-way analysis of variance with Tukey's *post-hoc* test (A, C, D, E, L).

**Figure S7**


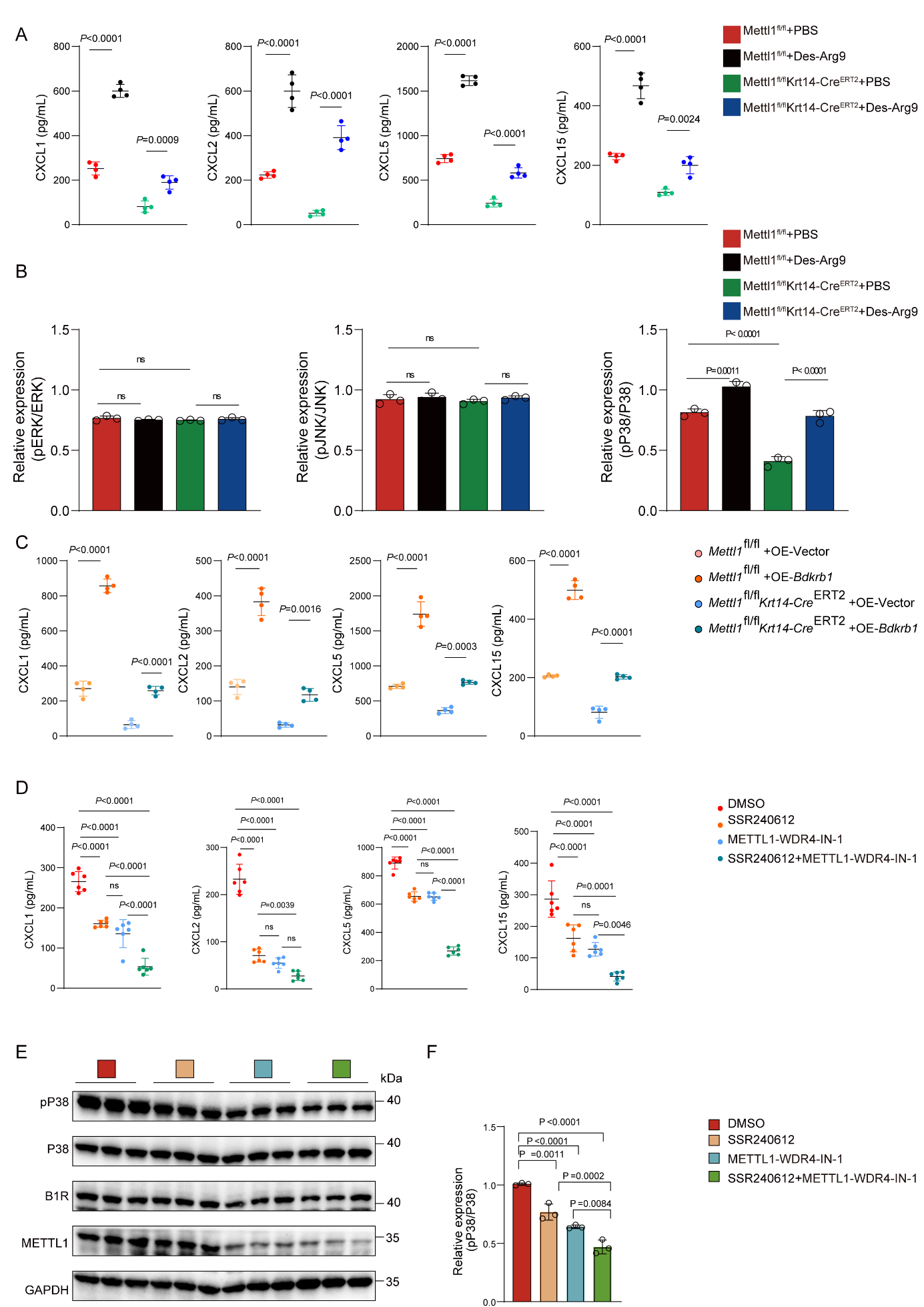


**Figure S7.** ***In vivo* validation of the METTL1–BDKRB1–p38 axis at the chemokine protein level.** (A) ELISA quantification of CXCL1, CXCL2, CXCL5, and CXCL15 protein levels in serum of IMQ-treated *Mettl1*fl/fl and *Mettl1*fl/fl*Krt14*-CreERT2 mice receiving PBS or the BDKRB1 agonist Des-Arg9 (n = 4 per group). (B) Quantification of the relative expression of pERK/ERK, pJNK/JNK, and pP38/P38 from Western blot analysis of the epidermis across the indicated treatment groups (n = 4 per group). (C) ELISA quantification of CXCL1, CXCL2, CXCL5, and CXCL15 protein levels in serum of IMQ-treated *Mettl1*fl/fl and *Mettl1*fl/fl*Krt14*-CreERT2 mice receiving K14-AAV9-Vector or K14-AAV9-OE-*Bdkrb1* (n = 4 per group). (D) ELISA quantification of CXCL1, CXCL2, CXCL5, and CXCL15 protein levels in serum of IMQ-treated mice receiving DMSO, SSR240612, METTL1-WDR4-IN-1, or their combination (n = 6 per group). (E, F) Western blot analysis (E) and quantification (F) of pP38, P38, B1R (BDKRB1), and METTL1 in the epidermis of mice across the indicated pharmacological treatment groups. GAPDH was used as the loading control (n = 3 per group). Data are representative of three independent experiments and shown as mean ± SD. Statistical significance was determined by one-way analysis of variance with Tukey's *post-hoc* test (A–D, F). ns, not significant.

**Supplementary Table**

**Table S1.** Sequences of siRNAs and shRNA

| Target | siRNA ID | Sense Strand (5'→3') | Antisense Strand (5'→3') |
| --- | --- | --- | --- |
| *NC* | siRNA-NC | UUCUCCGAACGUGUCACGU TT | ACGUGACACGUUCGGAGAA TT |
| *Bdkrb1* | siRNA-1 | CAAGGAUUGUGGAGUUAAATT | UUUAACUCCACAAUCCUUGTT |
|  | siRNA-2 | CGAUCGUCUUCUUCAACUATT | UAGUUGAAGAAGACGAUCGTT |
|  | siRNA-3 | GGCCAAUUUGUUCAUCAGCAU | GCUGAUGAACAAAUUGGCCUU |
| *DGKG* | siRNA-1 | CAGCGCAGAUACUAAUAUATT | UAUAUUAGUAUCUGCGCUGTT |
|  | siRNA-2 | GGAUUGCAUUGUCAACCAATT | UUGGUUGACAAUGCAAUCCTT |
|  | siRNA-3 | GGACCAUGAAGCACUUCAATT | UUGAAGUGCUUCAUGGUCCTT |
| *GPR84* | siRNA-1 | GCUAUCGUUAUGUUGCAGUTT | ACTGCAACATAACGATAGCTT |
|  | siRNA-2 | CCCUUCUUGCUGCUCAACATT | TGTTGAGCAGCAAGAAGGGTT |
|  | siRNA-3 | GCUAAGCAGAUGGCAGAGATT | TCTCTGCCATCTGCTTAGCTT |
| *MMP10* | siRNA-1 | GCCUAAGGUUGAUGCUGUA | UACAGCAUCAACCUUAGGC |
|  | siRNA-2 | GUACAAGCAGGUUAUCCAA | UUGGAUAACCUGCUUGUAC |
|  | siRNA-3 | GGACAGUAAUCUCAUUGUU | AACAAUGAGAUUACUGUCC |
| *BTNL2* | siRNA-1 | GACGUCACUUGUUCCAUCA | UGAUGGAACAAGUGACGUC |
|  | siRNA-2 | GAAAGACGAUACCAUCCUC | GAGGAUGGUAUCGUCUUUC |
|  | siRNA-3 | CACGUGCAGACAUUGCUAA | UUAGCAAUGUCUGCACGUG |
| *Rgs7bp* | siRNA-1 | CGUAGAUAGUCAGCAACAU | AUGUUGCUGACUAUCUACG |
|  | siRNA-2 | CCAAGAGUUUGGAUUGCAA | UUGCAAUCCAAACUCUUGG |
|  | siRNA-3 | GCUGUGUUGUCUCAUCUCA | UGAGAUGAGACAACACAGC |
| *SLC13A3* | siRNA-1 | CCGAUAACCAGCAGUCAGA | UCUGACUGCUGGUUAUCGG |
|  | siRNA-2 | GAAUAUCGUCGGAACAUCU | AGAUGUUCCGACGAUAUUC |
|  | siRNA-3 | GAAUCGCCCUCAAGAUCCU | AGGAUCUUGAGGGCGAUUC |
| shRNA | | | |
| *Mettl1* | Top strand | GATCCGGTGTATACCATAACCGATGTCTCGAGACATCGGTTATGGTATACACCTTTTTTG | |
|  | Bottom strand | AATTCAAAAAAGGTGTATACCATAACCGATGTCTCGAGACATCGGTTATGGTATACACCG | |

**Table S2.** Primer sequence of Quantitative PCR

| Gene Name | Species | Forward Sequence (5'→3') | Reverse Sequence (5'→3') |
| --- | --- | --- | --- |
| *GAPDH* | human | GGAGCGAGATCCCTCCAAAAT | GGCTGTTGTCATACTTCTCATGG |
| *METTL1* | human | GGCAACGTGCTCACTCCAA | CACAGCCTATGTCTGCAAACT |
| *IL1B* | human | ATGATGGCTTATTACAGTGGCAA | GTCGGAGATTCGTAGCTGGA |
| *IL6* | human | ACTCACCTCTTCAGAACGAATTG | CCATCTTTGGAAGGTTCAGGTTG |
| *IL17A* | human | TCCCACGAAATCCAGGATGC | GGATGTTCAGGTTGACCATCAC |
| *IL17F* | human | GCTGTCGATATTGGGGCTTG | GGAAACGCGCTGGTTTTCAT |
| *IL23A* | human | CTCAGGGACAACAGTCAGTTC | ACAGGGCTATCAGGGAGCA |
| *CAMP* | human | AGGTCCTCAGCTACAAGGAAG | TCTTGAAGTCACAATCCTCTGGT |
| *CCL2* | human | CAGCCAGATGCAATCAATGCC | TGGAATCCTGAACCCACTTCT |
| *CCL20* | human | TGCTGTACCAAGAGTTTGCTC | CGCACACAGACAACTTTTTCTTT |
| *TNF* | human | CCTCTCTCTAATCAGCCCTCTG | GAGGACCTGGGAGTAGATGAG |
| *S100A8* | human | ATGCCGTCTACAGGGATGAC | ACTGAGGACACTCGGTCTCTA |
| *S100A9* | human | GGTCATAGAACACATCATGGAGG | GGCCTGGCTTATGGTGGTG |
| *CXCL1* | human | CAATCCTGCACCCCAATAGT | GGATTTCACGTGTACGCACTTT |
| *CXCL2* | human | AGATCAATGTGACCGCAGGG | TCTCTGCTCTAACACAGAGGGA |
| *CXCL3* | human | AAAGGATACTGAACAGGGGAGCA | CTCTGGTAAGGGGCAGGGACC |
| *CXCL5* | human | AGCTGCGTTGCGTTTGTTTAC | TGGCGGAACACTTCGAGATTAC |
| *CXCL6* | human | AGAGCTGGGTTGCACCTTTT | GGACTTTTACCAATCGTTTTGGGG |
| *CXCL8* | human | AACTGAGAGTGATTGAGAGTGG | ATGAATTCTCAGCCCTCTTCAA |
| *Gapdh* | mouse | AGGTCGGTGTGAACGGATTTG | TGTAGACCATGTAGTTGAGGTCA |
| *Mettl1* | mouse | CAGACCACACACTGCGCTA | CATCCTTTGGATCATCATGGCTC |
| *Il1b* | mouse | GCAACTGTTCCTGAACTCAACT | ATCTTTTGGGGTCCGTCAACT |
| *Il5* | mouse | CTCTGTTGACAAGCAATGAGACG | TCTTCAGTATGTCTAGCCCCTG |
| *Il6* | mouse | TAGTCCTTCCTACCCCAATTTCC | TTGGTCCTTAGCCACTCCTTC |
| *Il12a* | mouse | CTGTGCCTTGGTAGCATCTATG | GCAGAGTCTCGCCATTATGATTC |
| *Il13* | mouse | CCTGGCTCTTGCTTGCCTT | GGTCTTGTGTGATGTTGCTCA |
| *Il15* | mouse | ACATCCATCTCGTGCTACTTGT | GCCTCTGTTTTAGGGAGACCT |
| *Il17a* | mouse | TTTAACTCCCTTGGCGCAAAA | CTTTCCCTCCGCATTGACAC |
| *Il18* | mouse | GACTCTTGCGTCAACTTCAAGG | CAGGCTGTCTTTTGTCAACGA |
| *Il19* | mouse | CTCCTGGGCATGACGTTGATT | GCATGGCTCTCTTGATCTCGT |
| *Il20* | mouse | TCTTGCCTTTGGACTGTTCTCC | GTTTGCAGTAATCACACAGCTTC |
| *Il22* | mouse | ATGAGTTTTTCCCTTATGGGGAC | GCTGGAAGTTGGACACCTCAA |
| *Il23a* | mouse | ATGCTGGATTGCAGAGCAGTA | ACGGGGCACATTATTTTTAGTCT |
| *Lcn2* | mouse | TGGCCCTGAGTGTCATGTG | CTCTTGTAGCTCATAGATGGTGC |
| *Ccl2* | mouse | TTAAAAACCTGGATCGGAACCAA | GCATTAGCTTCAGATTTACGGGT |
| *Ccl20* | mouse | GCCTCTCGTACATACAGACGC | CCAGTTCTGCTTTGGATCAGC |
| *TNF* | mouse | CCCTCACACTCAGATCATCTTCT | GCTACGACGTGGGCTACAG |
| *S100a8* | mouse | AAATCACCATGCCCTCTACAAG | CCCACTTTTATCACCATCGCAA |
| *S100a9* | mouse | ATACTCTAGGAAGGAAGGACACC | TCCATGATGTCATTTATGAGGGC |
| *Cxcl1* | mouse | CTGGGATTCACCTCAAGAACATC | CAGGGTCAAGGCAAGCCTC |
| *Cxcl2* | mouse | CCAACCACCAGGCTACAGG | GGCTCACACTCAAGCTCTG |
| *Cxcl3* | mouse | CCAGAGCAGAGACCTTATCCAC | CTTCATCATGGTGAGGGGTT |
| *Cxcl5* | mouse | TCCAGCTCGCCATTCATGC | TTGCGGCTATGACTGAGGAAG |
| *Cxcl15* | mouse | TGTTGAGCATGAAAGGCCTTAT | AGGTCTCCGGAATTGGAAGG |
